# Efficacy of Shenkang Injection combined with renin-angiotensin-aldosterone system blockers in diabetic nephropathy: a systematic review and meta-analysis of randomized controlled trials

**DOI:** 10.1080/0886022X.2025.2499231

**Published:** 2025-05-27

**Authors:** Xinyue Zhang, Xuyuan Lao, Shengchun Liao, Chaoyang Ye, Chen Wang

**Affiliations:** ^a^ Department of Nephrology, Shuguang Hospital Affiliated to Shanghai University of Traditional Chinese Medicine, Shanghai, China; ^b^ Department of Cardiology, Shuguang Hospital Affiliated to Shanghai University of Traditional Chinese Medicine, Shanghai, China; ^c^ Key Laboratory of Liver and Kidney Diseases, Ministry of Education, Shanghai University of Traditional Chinese Medicine, Shanghai, China; ^d^ TCM Institute of Kidney Disease, Shanghai University of Traditional Chinese Medicine, Shanghai, China; ^e^ Shanghai Key Laboratory of Traditional Chinese Clinical Medicine, Shanghai University of Traditional Chinese Medicine, Shanghai, China

**Keywords:** Shenkang Injection, Renin-angiotensin-aldosterone system (RAAS) blockers, traditional Chinese medicine, diabetic nephropathy, systematic review, meta-analysis

## Abstract

**Objective:**

Shenkang injection (SKI), a Traditional Chinese Medicine formulation, is widely used in China for diabetic nephropathy (DN). This systematic review and meta-analysis aimed to evaluate the efficacy and safety of SKI combined with renin-angiotensin-aldosterone system (RAAS) blockers in patients with DN.

**Methods:**

A comprehensive search of seven databases was conducted up to September 18, 2024, for randomized controlled trials (RCTs) comparing SKI plus RAAS blockers versus RAAS blockers alone in DN patients. Meta-analysis was performed using RevMan 5.3 and Stata 17.0, with effect sizes expressed as weighted mean differences (WMDs) or odds ratios (ORs) with 95% confidence intervals (CIs).

**Results:**

A total of 18 RCTs involving 1,497 patients were analyzed. Combination therapy significantly improved total effective rate (TER) (MD 2.61, 95% CI 1.62–2.64) and reduced key renal and metabolic markers. Urinary protein excretion rate (UPER), serum creatinine (SCr), blood urea nitrogen (BUN), 24-hour urinary protein (24h-UTP), total cholesterol (TC), and triglyceride (TG) levels all significantly decreased in the combination group. Subgroup analysis showed that patients aged ≤55 years had greater reductions in SCr (WMD −25.62, 95% CI −29.41 to −21.83) and BUN (WMD −2.51, 95% CI −2.75 to −2.27). Sensitivity analysis confirmed the robustness of findings. No publication bias was detected for TER, SCr, BUN, 24h-UTP, TG, and adverse reactions, though UPER and TC showed potential bias.

**Conclusion:**

SKI combined with RAAS blockers may enhance renal function and metabolic profiles in DN patients. Further high-quality RCTs are needed to validate these findings and assess long-term safety.

## 1. Introduction

Diabetic nephropathy (DN) is a major microvascular complication of diabetes mellitus [1]. A significantly elevated urinary protein excretion rate (UPER) is a hallmark of DN, and without timely intervention, progressive proteinuria can ultimately lead to renal failure. Diabetic nephropathy (DN) has become the leading cause of end-stage renal disease (ESRD) worldwide, recent epidemiology suggests that approximately 40% of patients with type 2 diabetes worldwide will develop DN, of which 30–50% will progress to ESRD [2]. Recent cohort studies have shown that the annual incidence of DN in patients with diabetes mellitus has increased from 2.1% in 2010 to 3.8% in 2022, with proteinuria-positive patients having up to a 28.7% risk of progression to ESRD within 5 years [3,4]. Renin-angiotensin-aldosterone system (RAAS) blockers, particularly angiotensin-converting enzyme inhibitors (ACEIs) and angiotensin receptor blockers (ARBs), are commonly used in clinical practice and have shown promising results in treating DN [5]. Recent systematic review highlighted their efficacy in DN: a meta-analysis of 46 studies (26,551 patients) confirmed RAAS blockers significantly reduce serum creatinine (mean difference: −13.4 μmol/L) and albuminuria compared to placebo [6]. However, RAAS blockade alone may not be sufficient to significantly reduce proteinuria in patients with severe proteinuria or reverse DN progression, highlighting the need for additional therapeutic strategies [7]. The latest guidelines for DN management recommend the use of combination therapies, which include RAAS blockers with sodium-glucose cotransporter 2 and mineralocorticoid receptor antagonist inhibitors [8].

Previous studies have confirmed that various types of TCM have significant improvement effects on DN, highlighting the potential of TCM in treating kidney disease [9]. Shenkang Injection (SKI), a standardized TCM injection manufactured by Xi’an Century Shengkang Pharmaceutical Co., Ltd. (Shaanxi, China), is widely used in China for kidney disease treatment. It is composed of four herbs, *Salvia miltiorrhiza, Safflower, Rhubarb, and Radix Astragali*, and is widely used in China for the treatment of kidney disease [10]. Pharmacological studies have shown that *Salvia miltiorrhiza* and *Safflower* have anticoagulant, platelet aggregation inhibitory and vascular endothelial damage attenuating effects, while *Rhubarb* and *Radix Astragali* have anti-inflammatory and antioxidant effects [11,12]. In TCM theory, the synergistic effect of *Salvia miltiorrhiza* and *Safflower* has the efficacy of activating blood circulation, removing blood stasis and improving hemodynamics, while *Rhubarb and Radix Astragali* act synergistically to benefit qi and drain turbidity and improve renal oxidative stress and fibrosis [13,14] This synergistic effect integrates hemodynamic regulation with anti-inflammatory and antioxidative pathways, collectively contributing to renal protection in DN.

Basic studies have confirmed that SKI exerts renoprotective effects in DN models by reducing oxidative stress, inflammation, and coagulation dysfunction, thereby delaying the progression of renal failure [15]. At the molecular level, SKI activates the Kelch-like ECH-associated protein 1 pathway, protecting against oxidative stress induced by hyperglycemic toxicity [16]. Additionally, several clinical studies have demonstrated SKI’s significant effects in treating DN, such as reducing urinary protein excretion rates (UPER), improving renal function, and mitigating the hypercoagulable state [17]. Given that RAAS blockers primarily target intraglomerular hypertension and proteinuria *via* hemodynamic modulation, while SKI acts through anti-inflammatory, antioxidant, and endothelial-protective mechanisms, their combination may exert complementary effects on multiple DN pathophysiological pathways. We hypothesize that the addition of SKI to RAAS blockers may enhance renal protection through addressing hemodynamic and anti-inflammatory mechanisms. Thus a systematic review and meta-analysis is warranted to provide a comprehensive evaluation of the efficacy and safety of SKI combined with RAAS blockers in DN treatment. This study includes randomized controlled trials (RCTs) assessing this combination therapy, aiming to provide high-quality evidence to support its clinical application.

## 2. Materials and methods

This systematic review aligns with the Preferred Reporting Items for Systematic Reviews and Meta Analyses (PRISMA 2020) guidelines [18]. The review was also preregistered prior to conducting the review: INPlASY, no. 202490042, DOI: 10.37766/inplasy2024.9.0042. This article is based on previously conducted research and does not contain any new research conducted by the authors on human participants or animals.

### 2.1. Search strategy

We systematically searched multiple databases, including PubMed, Cochrane Library, Embase, CNKI, CBM, WanFang Data, and VIP, for articles published between September 2004 and September 2024. While we did not include specific gray literature databases in our search., we manually reviewed the references of the included studies to identify any relevant studies that may have been missed during the initial search.

In the English-language databases, we applied the following search terms: “Diabetic nephropathy” OR “Diabetic kidney disease” in combination with “Shenkang” OR “Shenkang injection,” along with “Renin-angiotensin-aldosterone system blockers” OR “RAAS blockers” OR “Angiotensin-converting enzyme inhibitors” OR “Angiotensin receptor blockers” AND “Randomized control.” In the Chinese-language databases, we used equivalent terms: “Tang Niao Bing Shen Bing” (diabetic nephropathy), paired with “Shen Kang” OR “Shen Kang Zhu She Ye” (SKI), and “RAAS Zu Zhi Ji” OR “Xue Guan Jin Zhang Su Zhuan Huan Mei Yi Zhi Ji” (RAAS blockers, ACEIs, and ARBs), combined with “sui ji” (randomized control). The titles and abstracts were initially screened, followed by a full-text review for inclusion and exclusion criteria. Two independent reviewers assessed study quality and extracted data, with a third reviewer consulted for discrepancies. We assessed inter-reviewer agreement using kappa (κ) statistics, with a κ value of 0.77 for study selection, indicating substantial agreement.

### 2.2. Inclusion criteria

Subjects: Patients with a definite diagnosis of DN who meet the KDIGO (Kidney Disease: Improving Global Outcomes) 2020 Clinical Practice Guideline for Diabetes Management [19]. Patients will be of any age, sex, race, or clinical stage.

Studies: The literature included in this study were RCTs with no restrictions on blinding or concealment of group allocation. There was no restriction on the type of literature, language or population characteristics.

Subgroups and interventions: Literature is included that compares the efficacy and safety of SKI in combination with RAAS blockers and RAAS blockers alone (ACEI or ARB) in the treatment of DN, irrespective of dose, type, or duration of treatment. Primary treatment for DN was similar in two groups.

Outcomes: The primary outcomes were the total effective rate (TER), UPER and 24 h urinary protein level (24h-UTP). Secondary outcomes comprised serum creatinine (SCr), blood urine nitrogen (BUN), total cholesterol (TC) and triglyceride (TG).

### 2.3. Exclusion criteria

Articles were excluded from the analysis for the following reasons: (1) trials that did not meet the predetermined inclusion criteria; (2) studies using herbal preparations other than SKI; (3) patients with other types of kidney disease; (4) studies based on animal models, *in vitro* experiments, systematic reviews, or conference papers; and (5) duplicate publications or articles with incomplete data.

### 2.4. Data extraction

Data from eligible literature were extracted independently by two researchers. In case of disagreements, a third researcher was involved to resolve discrepancies. Initially, the titles and abstracts of the retrieved articles were screened using EndNote X9 software to exclude studies that did not meet the criteria. Then, the full text of the articles was thoroughly assessed to confirm whether they met the inclusion criteria. The collected data were categorized by author (year), sample size, interventions, comparators, treatment duration, outcome assessment, and observed adverse events. To maintain uniformity, outcome measures reported in different units were converted using standardized conversion factors. The characteristics of the included studies and clinical outcome data were summarized and presented in tabular form. Data extraction was also independently evaluated by two reviewers, with a third reviewer consulted in cases of discrepancies. We also assessed inter-reviewer agreement with a κ value of 0.82 for data extraction, indicating almost perfect agreement.

### 2.5. Quality assessment

We assessed research bias using the ‘Revised tool to assess risk of bias in randomized trials’ (RoB 2) [20]. Each study was classified as having either ‘high risk of bias,’ ‘low risk of bias,’ or ‘some concerns’ based on these criteria. The overall quality of evidence for each primary outcome was evaluated using the Grading of Recommendations Assessment, Development, and Evaluation (GRADE) system [21]. This assessment rated the evidence across four levels (‘very low,’ ‘low,’ ‘moderate,’ and ‘high’) by examining factors such as risk of bias, inconsistency, indirectness, imprecision, and potential publication bias. Two reviewers independently evaluated the risk of bias and the methodological quality, with a third reviewer involved to resolve any disagreements.

### 2.6. Statistical analysis

All data were analyzed using RevMan 5.3 and Stata 17.0. For dichotomous variables, we used odds ratio (OR) and 95% confidence interval (CI). For continuous variables, we used weighted mean difference (WMD) and 95% CI. Success rates (95% CI) and results of pooled analyses for each study are shown as forest plots. Heterogeneity was assessed using I^2^ statistics to quantify the proportion of variability due to heterogeneity and τ^2^ (Tau-squared) as an absolute measure of variance across studies. A random-effects model was applied when substantial heterogeneity was detected. The weighting of the study was based on the DerSimonian and Laird stochastic models and is shown in the form of a forest plot. Knapp-Hartung adjustment was applied to improve the robustness of the standard error estimation and to provide more reliable confidence intervals. A funnel plot was used to represent the publication bias analysis, and the Egger and Begger test was added to quantify the extent of publication bias. Subgroup analyses were also added to the basic analyses to determine differences in efficacy in patients with DN at different ages. Finally, sensitivity analyses were used to determine the stability of the meta-analysis results.

## 3. Results

### 3.1. Search results

**Figure 1.**
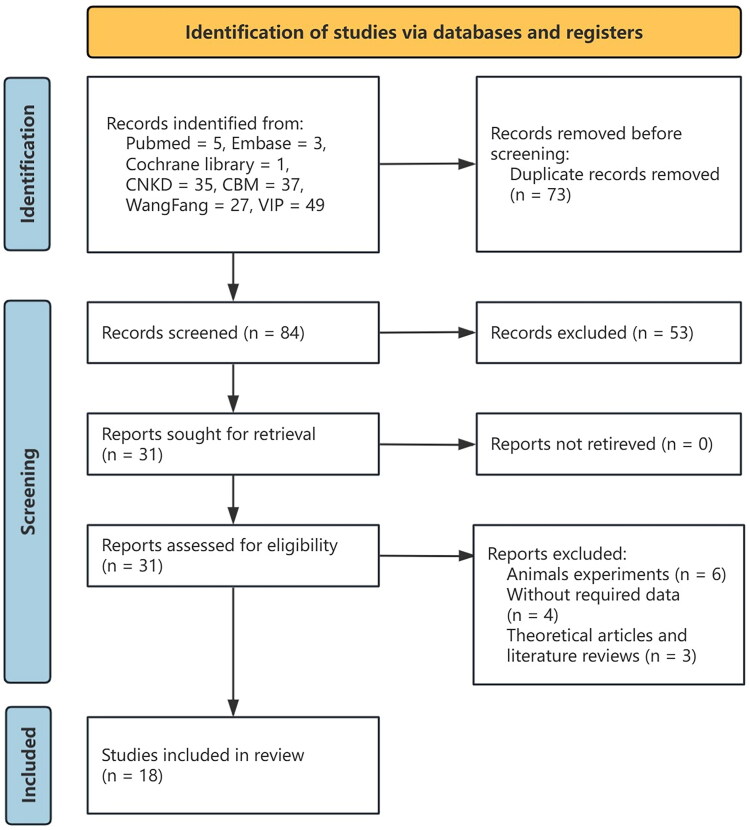
Flow chart of article selection.

A total of 157 references were retrieved through the database. After removing 73 duplicates, the abstracts of 84 were screened for eligibility, 41 studies unrelated to the study topic were excluded, and the remaining 31 were thoroughly assessed. Ultimately, 18 randomized controlled trials were included in this meta-analysis [22–39]. Figure 1 shows the literature search process and study selection for this study. The study sample included a total of 1497 subjects, of which 747 received combination therapy (treatment group) and 150 received only RAAS blockers (control group), with a minimum treatment duration of 2 weeks and a maximum of 12 weeks in each group. Table 1 shows the main characteristics of the included studies.

**Table 1.** Characteristics of included studies.

Author	Participants (Trial/Control)	Intervention		Course (weeks)	Report outcomes
		Combination therapy	Control		
Hao 2010 [22]	21/21	SKI injection (100 ml/d) + Irbesartan	Irbesartan	9	①②⑥⑦
Zhang 2012 [23]	20/23	SKI injection (60 ml/d) + Fosinopril	Fosinopril	2	②③④
Ma 2013 [24]	41/41	SKI injection (60 ml/d) + Benazepril	Benazepril	8	③④⑤⑥⑦
Xiao 2013 [25]	34/34	SKI injection (100 ml/d) + Losartan	Losartan	8	⑤⑥⑦
Wang 2013 [26]	42/42	SKI injection (100 ml/d) + Irbesartan	Irbesartan	12	①②③④
Li 2015 [27]	30/30	SKI injection (60 ml/d) + Olmesartan	Olmesartan	4	②③④⑧
Hu 2015 [28]	36/36	SKI injection (100 ml/d) + Telmisartan	Telmisartan	4	②③④
Han 2016 [29]	60/60	SKI injection (100 ml/d) + Irbesartan	Irbesartan	9	②⑤⑥⑦
Bao2016 [30]	47/47	SKI injection (100 ml/d) + Telmisartan	Telmisartan	4	①②③④⑤
Wu 2016 [31]	30/30	SKI injection (100 ml /d) + Irbesartan	Irbesartan	4	①②
Ren 2018 [32]	50/50	SKI injection (100 ml /d) + Irbesartan	Irbesartan	3	①②③④
Tao 2019 [33]	30/30	SKI injection (100 ml /d) + Candesartan	Candesartan	4	①②③④⑥⑦
Jiang 2019 [34]	30/30	SKI injection (100 ml /d) + Valsartan	Valsartan	4	①②③④
Deng 2021 [35]	91/91	SKI injection (60 ml /d) + Valsartan	Valsartan	12	①③④⑤
Yan 2021 [36]	60/60	SKI injection (100 ml /d) + Irbesartan	Irbesartan	12	①②
Liang 2021 [37]	35/35	SKI injection (60 ml /d) + Valsartan	Valsartan	4	①③④⑤
Li 2022 [38]	44/44	SKI injection (60 ml /d) + Irbesartan	Irbesartan	12	②③④⑥⑦
Wu 2023 [39]	46/46	SKI injection (100 ml /d) + Losartan	Losartan	12	①⑤

**Abbreviations:** ① TER: total effective rate; ② UPER: urinary protein excretion rate; ③ SCr: serum creatinine; ④ Bun: blood urine nitrogen; ⑤ 24h-UTP: 24 h urinary protein level; ⑥ TC: total cholesterol; ⑦ TG: triglyceride.

### 3.2. Quality evaluation

**Figure 2.**
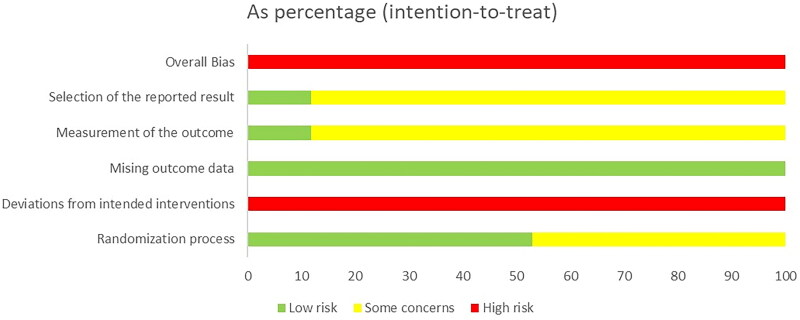
Risk bias of summary. Red, high risk; green, low risk; yellow, unclear risk.

Based on RoB 2, eight studies applied the random number table method for random sequence generation, classifying them as having a low risk of bias in this aspect. The other studies, lacking detailed descriptions of their random sequence generation methods, were approached with caution. None of the articles mentioned allocation concealment or blinding, placing them at high risk of bias. Despite using relatively objective measures, the absence of clarity on whether the studies were double-blind introduces some bias. Additionally, no pre-specified analytical protocols were identified in the included literature. A summary of the overall quality assessment is presented in Figure 2.

### 3.3. Report outcomes

#### 3.3.1. Total effective rate (TER)

**Figure 3.**
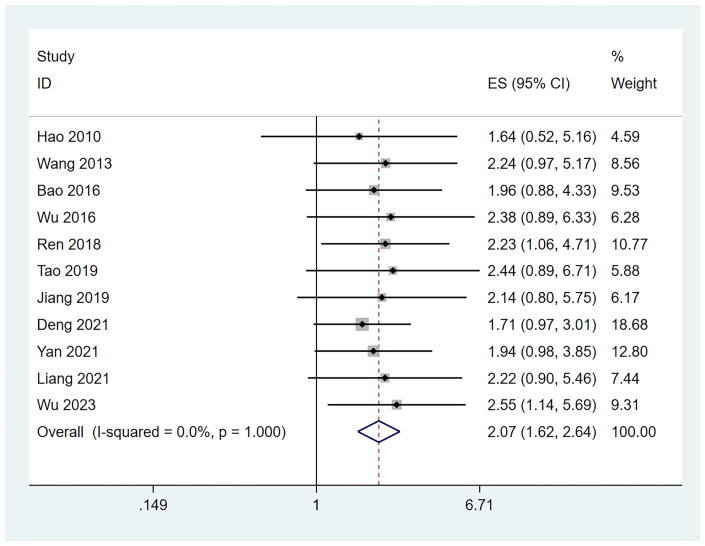
Forrest plot of TER. ES: effect size; CI: confidence interval.

A total of eleven studies involving 964 patients reported TER [24,26,30–37,39]. A fixed-effects model was applied due to the low heterogeneity. The combination group showed significantly higher clinical efficacy than the RAAS blockers group (Figure 3 and Table 2).

**Table 2.** Summary of primary outcomes comparing SKI combined with RAAS blockers versus RAAS blockers alone in DN patients.

Outcome	number of articles	Sample size (Trial/Control)	Effect size (WMD/OR)	95% CI	I² (%)	*p*	τ²
TER	11	482 / 482	OR = 2.61	[1.65, 2.55]	0.00	<0.001	0.00
UPER	19	500 / 503	WMD = −25.81	[–28.34, −23.28]	17.9	<0.001	4.36
SCr	12	496 / 499	WMD = −16.77	[–20.36, −13.19]	75.3	<0.001	22.51
BUN	12	496 / 499	WMD = −1.46	[–1.89, −1.04]	90.0	<0.001	0.40
24h-UTP	6	294 / 294	WMD = −0.34	[–0.40, −0.28]	0.00	<0.001	0.00
TC	6	230 / 230	WMD = −0.18	[–0.23, −0.13]	12.4	<0.001	0.00
TG	6	230 / 230	WMD = −0.94	[–0.99, −0.88]	16.0	<0.001	0.00
Adverse Reaction	7	16 / 26	OR = −1.04	[0.83, 1.29]	0.00	0.759	0.00

**Abbreviations:** TER: total effective rate; UPER: urinary protein excretion rate; SCr: serum creatinine; Bun: blood urine nitrogen; 24h-UTP: 24 h urinary protein level; TC: total cholesterol; TG: triglyceride; OR: odds ratio; CI: confidence interval; WMD: weighted mean difference.

#### 3.3.2. Urinary protein excretion rate (UPER)

**Figure 4.**
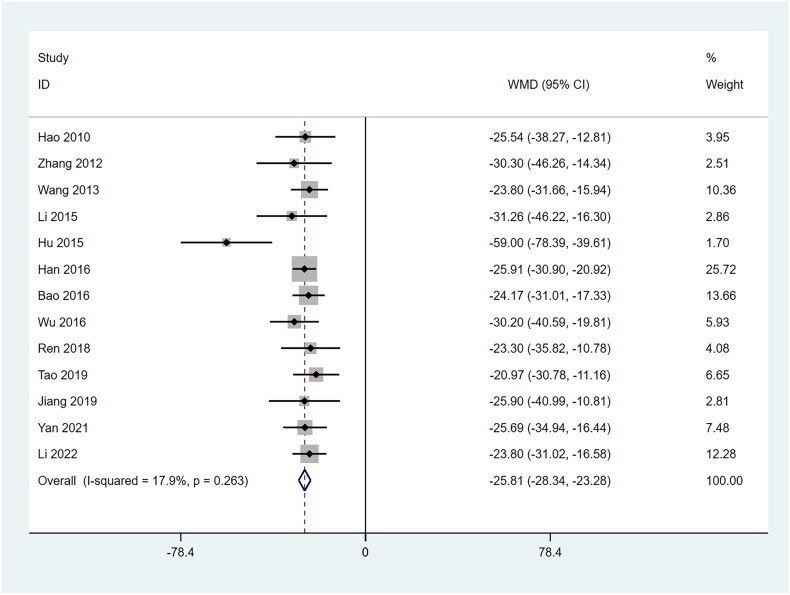
Forrest plot of UPER. WMD: weighted mean difference; CI: confidence interval.

Nineteen studies reported UPER, comprising 1003 patients [22,23,26–34,36,38]. A fixed-effects model was used. The combination therapy group exhibited significantly reduced proteinuria compared to control (Figure 4 and Table 2).

#### 3.3.3. Serum creatinine (SCr) and blood urine nitrogen (BUN)

Twelve studies reported SCr and BUN, including 995 patients [23,24,26–28,30,32–35,37,38]. Significant improvements were observed in both markers in the combination group. Due to substantial heterogeneity, a random-effects model was used (Figures S1 and S2, Table 2).

The aim of this study was to identify sources of heterogeneity in the included studies through subgroup analyses. Heterogeneity may arise due to differences in age, disease duration, and drug dosage of the patients included in the study, as well as differences in the timing and measures of the intervention. Subgroup analysis based on average age showed greater improvements in renal function (SCr and BUN) in patients aged ≤55 years than those >55 years, indicating that younger patients may respond better to combination therapy (Table 3).

**Table 3.** Subgroup analysis of SCr and BUN.

Outcomes	number of articles	Heterogeneity		Effects model	Effects value		
		I^2^	*p*		WMD	95% CI	*p*
SCr	12						
Average age ≤55 group	4	18%	0.301	Fixed	−25.62	[–29.41, −21.83]	<0.001
Average age > 55 group	8	48%	0.060	Fixed	−16.49	[–18.10, −14.88]	<0.001
BUN	12						
Average age ≤55 group	4	0%	0.630	Fixed	−2.51	[–2.75, −2.27]	<0.001
Average age > 55 group	8	53%	0.039	Fixed	−1.12	[–1.27, −0.98]	<0.001

**Abbreviations:** SCr: serum creatinine; Bun: blood urine nitrogen; CI: confidence interval; WMD: weighted mean difference.

#### 3.3.5. 24 h Urinary protein level (24h-UTP)

Six studies used 24h-UTP as a reporting indicator for urinary protein in the results [24,25,30,35,37,39], comprising 588 patients. A fixed-effects model was applied due to low heterogeneity. Results favored the combination group (Figure S3 and Table 2).

#### 3.3.6. Total cholesterol (TC) and triglyceride (TG)

Six eligible studies have reported lipid indicators (TC and TG), including 460 patients [22,24,25,29,33,38]. A fixed-effects model was used. Both TC and TG were significantly lower in the combination group (Figures S4 and S5, Table 2).

**Figure 5.**
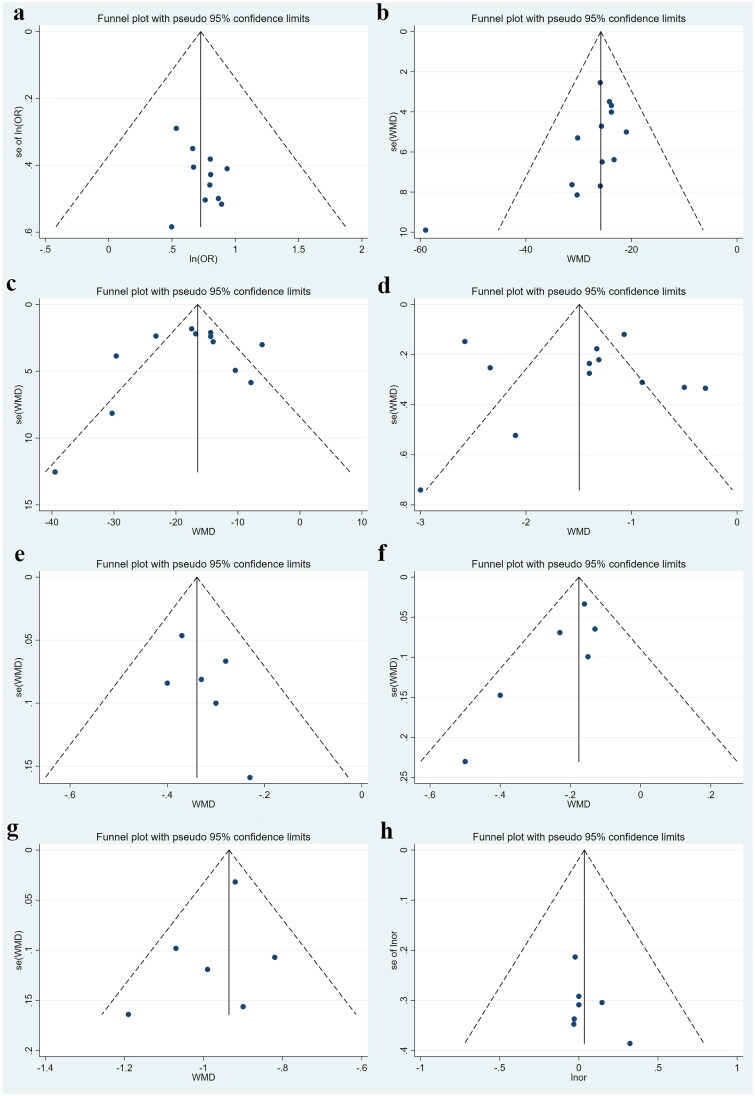
Funnel plots for TER, UPER, SCr, BUN, 24h-UTP, TC, TG and adverse reaction. (a) TER; (b) UPER; (c) SCr; (d) BUN; (e) 24h-UTP; (f) TC; (g) TG; (h) adverse reaction. SE: Standard Error; OR: odds ratio difference; WMD: weighted mean difference.

#### 3.3.7. Adverse reaction

A total of seven studies [26,28,30,31,35,37,39] reported adverse reactions, including dizziness, nausea, and diarrhea, in 42 patients. A fixed-effects model was applied. No significant differences were observed between groups (Figure S6 and Table 2).

#### 3.3.8. Publication bias and sensitivity analyses

A total of seven clinical indicators were included in the results of the publication bias analysis. Publication bias was quantitatively assessed using Egger’s test and Begg’s test.The results related to TER, UPER, SCr, BUN, 24h-UTP, TC, TG and adverse reaction were not significantly different (*p* > 0.05, Table 4). Further observation of the funnel plots showed that the funnel plots of TER, SCr, BUN, 24h-UTP, TG and adverse reaction were basically symmetrical, whereas the funnel plots of UPER and TC were less symmetrical (Figure 5). This suggests that there is no publication bias in the meta-analysis of TER, SCr, BUN, 24h-UTP, TG and adverse reaction, whereas UPER and TC may have some risk of bias. It is worth noting that the literature included in the publication bias analysis were all small, of which only six were included in the analysis of TC. The small sample size of the included studies could also affect the results of the publication bias analysis.

**Table 4.** Publication bias.

Report outcomes	Number of articles (n)	Egger test		Begg test	
		t	*p*	t	*p*
TER	11	1.59	0.146	0.78	0.436
UPER	13	−1.93	0.080	1.89	0.059
SCr	12	−0.53	0.611	0.07	0.945
BUN	12	0.15	0.885	0.837	0.21
24h-UTP	6	1.25	0.280	0.75	0.452
TC	6	−2.20	0.093	1.50	0.133
TG	6	−0.98	0.381	0.75	0.452
Adverse Reaction	7	1.34	0.239	0.60	0.548

**Abbreviations:** TER: total effective rate; UPER: urinary protein excretion rate; SCr: serum creatinine; Bun: blood urine nitrogen; 24h-UTP: 24 h urinary protein level; TC: total cholesterol; TG: triglyceride.

Sensitivity analysis was used to assess the stability of the study results. Random effects and fixed effects models were used to comparatively analyze the reported outcomes in the study, respectively. The combined MD and 95% CI results were similar for both models (Table 5), demonstrating the stability of our study.

**Table 5.** Sensitivity analysis.

Report outcomes	Fixed effects medel			Random effects model		
	WMD	95% CI	*p*	WMD	95% CI	*p*
TER	2.61	[1.62, 2.64]	<0.001	2.61	[1.62, 2.64]	<0.001
UPER	−25.81	[–28.34, −23.28]	<0.001	−26.01	[–28.95, −23.07]	<0.001
SCr	−16.49	[–18.10, −14.88]	<0.001	−16.77	[–20.36, −13.19]	<0.001
BUN	−1.50	[–1.62, −1.37]	<0.001	−1.46	[–1.89, −1.04]	<0.001
24h-UTP	−0.34	[–0.40, −0.28]	<0.001	−0.34	[–0.40, −0.28]	<0.001
TC	−0.18	[–0.23, −0.13]	<0.001	−0.18	[–0.24, 0.12]	<0.001
TG	−0.94	[–0.99, −0.88]	<0.001	−0.95	[–1.02, −0.87]	<0.001
Adverse Reaction	1.04	[0.83, 1.29]	0.759	1.04	[0.83, 1.29]	0.759

**Abbreviations:** TER: total effective rate; UPER: urinary protein excretion rate; SCr: serum creatinine; Bun: blood urine nitrogen; 24h-UTP: 24 h urinary protein level; TC: total cholesterol; TG: triglyceride.

#### 3.3.9. GRADE evaluation of evidence quality

We used the GRADE system to categorize the quality of the key outcome indicators of the 18 papers according to high, medium, and low (Table 6). According to the GRADE evaluation, the quality of evidence for TER, SCr, BUN, 24h-UTP, TG and adverse reaction was moderate, and the quality of evidence for UPER and TC was low. All studies that included indicators did not describe how blinding and allocation concealment were implemented; SCr and BUN showed high heterogeneity but more robust results; and UPER and TC may be subject to publication bias. This needs to be further ­confirmed from more large-scale, multicenter RCTs.

**Table 6.** GRADE evaluation of evidence quality.

Report outcomes & included studies (n)	Limitations	Inconsistency	Indirectness	Imprecision	Publication bias	Participants (Trial/Control)	Effect Size (95% CI)	Quality
**TER** 11	Yes①	No	No	No	No	482/482	2.61 [1.62, 2.64]	Moderate
**UPER** 13	Yes①	No	No	No	Yes③	500/503	−25.81 [–28.34, −23.28]	Low
**SCr** 12	Yes①	Yes②	No	No	No	496/499	−16.77 [–20.36, −13.19]	Moderate
**BUN** 12	Yes①	Yes②	No	No	No	496/499	−1.46 [–1.89, −1.04]	Moderate
**24h-UTP** 6	Yes①	No	No	No	No	294/294	−0.34 [–0.40, −0.28]	Moderate
**TC** 6	Yes①	No	No	No	Yes③	230/230	−0.18 [–0.23, −0.13]	Low
**TG** 6	Yes①	No	No	No	No	230/230	−0.94 [–0.99, −0.88]	Moderate
**Adverse reaction** 7	Yes①	No	No	No	No	327/327	1.04 [0.83, 1.29]	Moderate

**Notes:** ① Included articles had a large bias in methodology, such as location concealment or blinding; ② I^2^ value of combined results was larger; ③ May lead to publication bias. **Abbreviations:** TER: total effective rate; UPER: urinary protein excretion rate; SCr: serum creatinine; Bun: blood urine nitrogen; 24h-UTP: 24 h urinary protein level; TC: total cholesterol; TG: triglyceride; CI: confidence interval.

## 4. Discussion

DN, one of the most common microvascular complications of diabetes mellitus, is clinically characterized by proteinuria, hypertension, and progressive renal function impairment, making it a leading cause of renal replacement therapy. The pathophysiology of DN is complex and multifactorial, involving both metabolic and hemodynamic factors. RAAS blockers, which reduce intraglomerular pressure and improve glycemic control and blood pressure management, are currently the first-line treatment for DN. However, current therapies cannot fully prevent the progression to end-stage renal disease in some patients [40]. This meta-analysis included 18 randomized controlled trials with a total of 1,497 patients to assess the clinical efficacy and safety of SKI combined with RAAS blockers for the treatment of DN.

Our findings suggest that: (1) The clinical efficacy of RAAS blockers in combination with SKIs is significantly better than that of RAAS blockers monotherapy. (2) Combination therapy may provide a reduction in urinary protein (UPER and 24-UTP) in patients with DN. (3) Combination therapy improves renal function (SCr and BUN) in patients with DN. (4) Combination therapy may have a lipid lowering effect (TC and TG) in patients with DN. (5) Seven papers specifically reported adverse reactions, and there were no serious adverse reactions. The difference in adverse reactions between the combination therapy group and the control group was not statistically significant. Although the Egger and Begg tests indicated no publication bias, the small number of studies included suggests that more high-quality research is needed to further confirm the safety of SKI. The pooled analysis in this study found that SKI combined with RAAS blockers was significantly more effective than ACEIs or ARBs alone in reducing proteinuria, improving renal function, and lowering lipids. This finding is consistent with Wang et al. [17]; however, unlike their meta-analysis, the present study specifically restricted ACEIs or ARBs—widely recognized as standard treatments for diabetic nephropathy—as the baseline medication, thereby minimizing potential confounding effects.However, variability in the included data, such as differences in patient age, disease duration, drug dosage regimens, timing of interventions, and measures taken, may have influenced the study’s outcomes. These potential effect modifiers could impact treatment outcomes, and further well-designed trials are needed to explore them.

Our further analyses showed that in subgroup analyses by age of included patients, the group of patients average age ≤ 55 group had a significantly better effect on both SCr and BUN than average age > 55 group. This suggests that patients with a lower mean age have better efficacy in improving renal function with SKIs in combination with RAAS blockers. Age is an independent risk factor for DN progression, with older patients exhibiting poorer renal function, lower hemoglobin and albumin levels, and a higher likelihood of developing ESRD [41]. Basic studies have further demonstrated that activation of the RAAS system is more pronounced and the development of DN is more rapid in the kidneys of aged rats with DN [42]. Additionally, the studies demonstrated stability through sensitivity analyses. However, according to the GRADE quality of evidence assessment, all studies ranged from low to moderate quality, which may have affected the overall reliability of this meta-analysis.

According to TCM theory, DN is primarily caused by qi and yin deficiency with blood stasis. Qi deficiency weakens regulation and leads to the loss of essence, resulting in proteinuria. Additionally, prolonged qi deficiency contributes to the development of blood stasis over time [43]. SKI is made from extracts of four herbs—*Salvia miltiorrhiza*, *Safflower*, *Rhubarb*, and *Radix Astragali*—which are known to benefit qi, nourish yin, invigorate blood circulation, promote diuresis, and eliminate turbidity and stasis [44].

Modern pharmacological studies have shown that *Astragalus* reduces proteinuria and improves pathological changes in DN, such as glomerular basement membrane thickening, thylakoid cell proliferation, and damage to endothelial cells, podocytes, and tubular cells [45]. *Rheum officinale* ameliorates glomerular injury in DN by stabilizing the podocyte cytoskeleton and correcting autophagy in damaged podocytes [46]. Extracts of *Salvia miltiorrhiza* and *Safflower* delay the progression of renal fibrosis in DN by modulating various signaling pathways, including metabolism, renal hemodynamics, oxidative stress, and inflammation [47,48]. Basic studies have shown that SKI reduces urinary albumin excretion, enhances renal function, and improves lipid metabolism in DN animal models, consistent with the results of our meta-analysis [49]. The mechanism of action of SKI may be related to its renal protective effects by inhibiting epithelial-mesenchymal transition and endoplasmic reticulum stress-induced apoptosis in diabetic renal tubular cells, thereby exerting antifibrotic effects [50].

Several limitations of this study should be considered. First, relevant grey literature was not included in this study, and the methodological quality of the included studies was generally low. Although all studies reported using randomized methods, none of the 18 studies implemented allocation concealment measures, raising concerns about the validity of the randomization process and potential selection bias. To mitigate this limitation, we conducted a comprehensive sensitivity analysis to assess the robustness of our findings and performed subgroup analyses to explore potential sources of heterogeneity. Second, all 18 studies were single-center trials with small sample sizes, while there are very few studies with missing data, which may have contributed to publication bias. To minimize the impact of this limitation, we assessed publication bias using funnel plot analysis and Egger’s test. Additionally, the dosage of SKI varied across studies, with treatment periods ranging from 2 to 12 weeks, and the lack of a placebo control group, along with multiple drug treatments, may not have allowed for a full assessment of the long-term efficacy and safety of the combination therapy. Therefore, future clinical trials should include placebo controls and comprehensive cost-effectiveness analyses. Moreover, given the evolving nature of DN treatment, future trials should incorporate sodium-dependent glucose transporter 2 inhibitors and mineralocorticoid receptor antagonists as control groups for a more comprehensive evaluation.

Despite these limitations, this study represents the first systematic assessment of the efficacy of SKI combined with RAAS blockers for the treatment of DN and may provide valuable insights for clinicians. Based on the findings of our meta-analysis, combination therapy may be particularly suitable for patients with moderate DN who continue to exhibit residual proteinuria despite standard RAAS blockers. Although exact dosing should be individualized based on patient characteristics, most included RCTs used intravenous SKI at doses of 20–40 mL per day, with treatment durations ranging from 4 to 12 weeks. Key monitoring parameters during treatment should include serum creatinine, urinary protein (UPER or 24h-UTP). Additionally, while some data suggest potential benefits in lipid metabolism, further evaluation is needed in larger trials. From a practical standpoint, broader clinical implementation may face challenges such as intravenous administration logistics and healthcare costs. Preliminary cost-effectiveness evidence remains limited and warrants further investigation.

Our findings may have important implications for clinical practice guidelines. The KDIGO 2022 guideline emphasizes RAAS blockers as the foundation of DN treatment but does not recommend any traditional herbal combination therapy [51]. In contrast, the Chinese guidelines for combined Chinese and Western medicine in 2024 suggest the potential of SKI as an adjunctive therapy to RAAS blockers to further reduce 24h-UTP and Scr, especially in patients with early and intermediate DN [52]. Our findings support this clinical practice pattern in China, showing that SKI combined with RAAS blockers improves renal function and reduces proteinuria more effectively than RAAS monotherapy. Although the included studies were rated as low to moderate in quality based on GRADE criteria, the consistent benefit across multiple outcomes suggests a potential role for SKI in guideline updates, especially within the context of real-world Chinese practice. However, the current evidence is not yet robust enough to change international recommendations. Moreover, SKI combination therapies may be more appropriate for patients with early to mid-stage DN and at a younger age who are at lower risk for these adverse effects. As the current study lacks stratification of disease severity, further evaluation of its role in each DN stage is needed.

This study demonstrated that SKI combined with RAAS blockers offers superior clinical efficacy, renal function improvement, lipid metabolism regulation, and proteinuria reduction compared to ACEI/ARB monotherapy, while maintaining a comparable safety profile. However, limitations exist in this meta-analysis, highlighting the need for further well-designed clinical trials to validate these findings. Future research should focus on optimizing dosage, treatment duration, and conducting long-term follow-up studies to assess sustained efficacy and safety.

## Supplementary Material

Figure documents.zip

Figure Supplementary documents.zip

## Data Availability

The datasets generated during and/or analyzed during the current study are available from the corresponding author on reasonable request.
