# Supplementary figures and images for: Efficacy of Shenkang Injection combined with renin-angiotensin-aldosterone system blockers in diabetic nephropathy: a systematic review and meta-analysis of randomized controlled trials
Source: Ren Fail. 2025 May 27;47(1):2499231. doi: 10.1080/0886022X.2025.2499231 (PMC12117859; doi:10.1080/0886022X.2025.2499231)

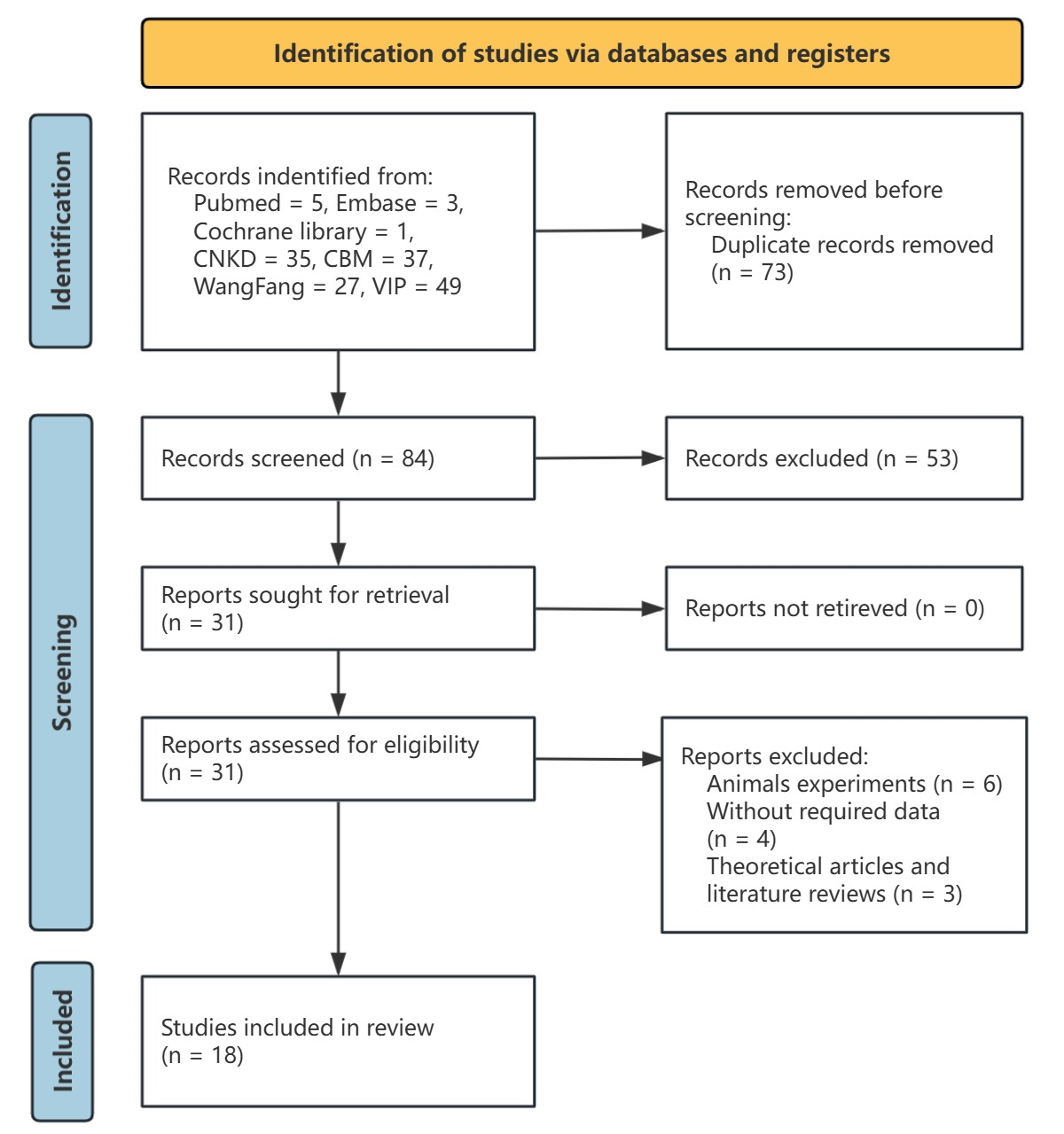

Supplement: Figure documents.zip [file IRNF_A_2499231_SM6438.zip › Figure 1.tif]

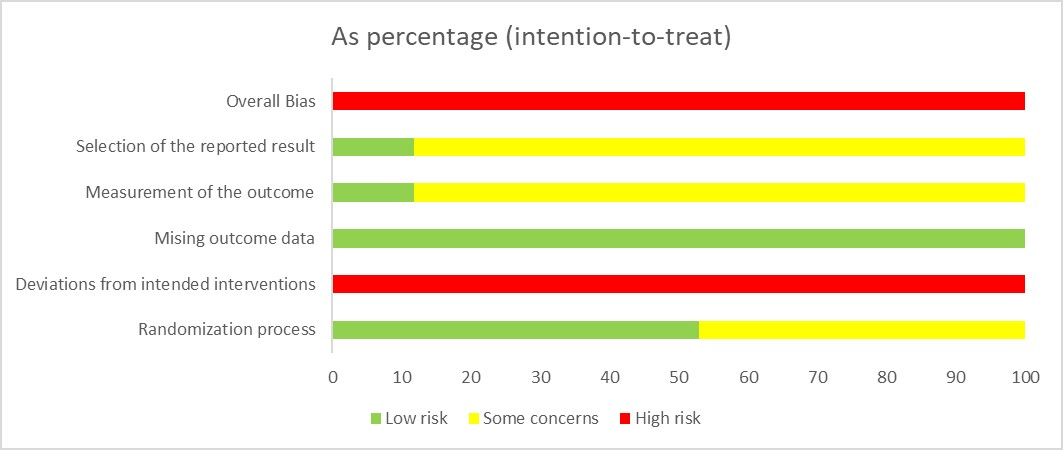

Supplement: Figure documents.zip [file IRNF_A_2499231_SM6438.zip › Figure 2.tif]

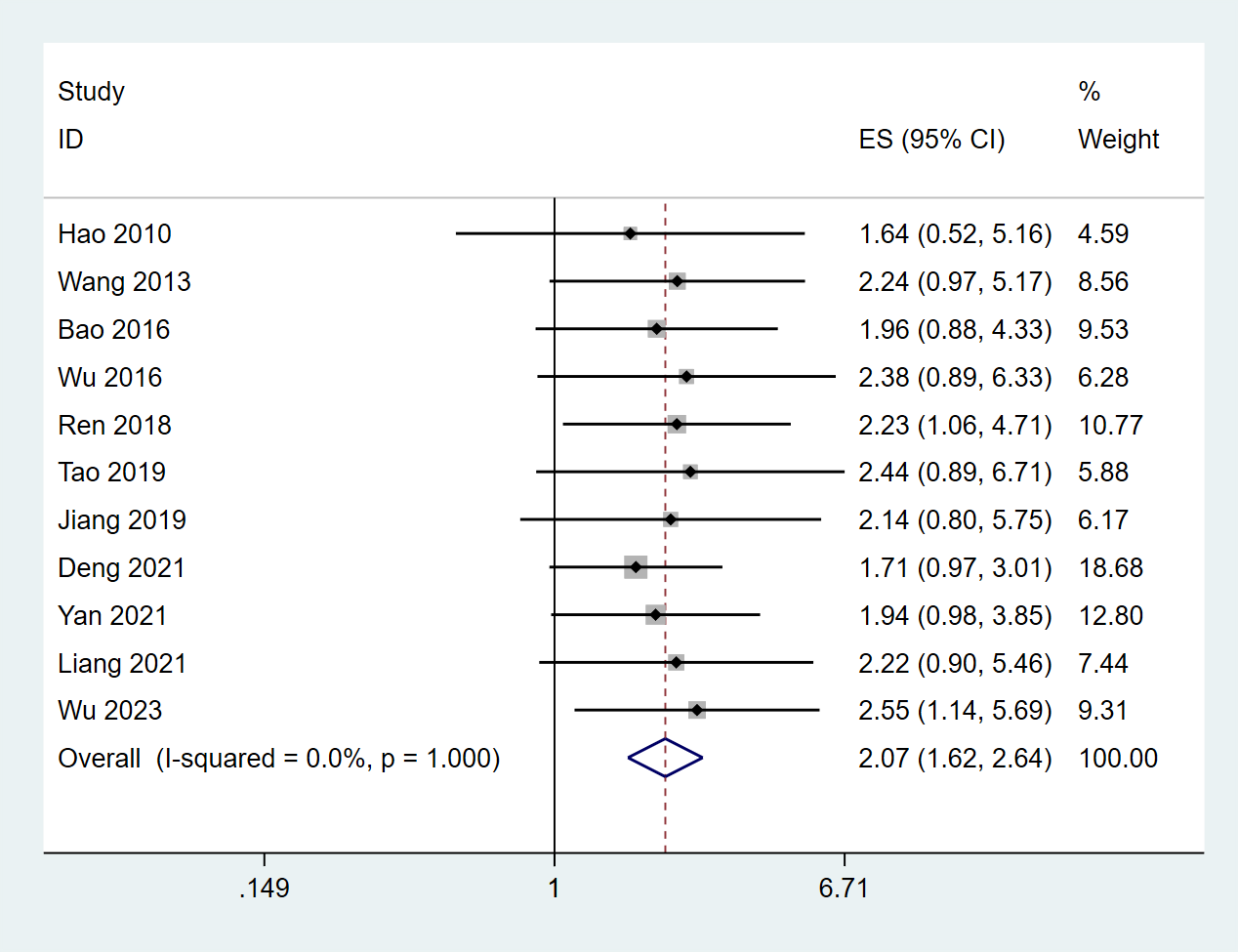

Supplement: Figure documents.zip [file IRNF_A_2499231_SM6438.zip › Figure 3.tif]

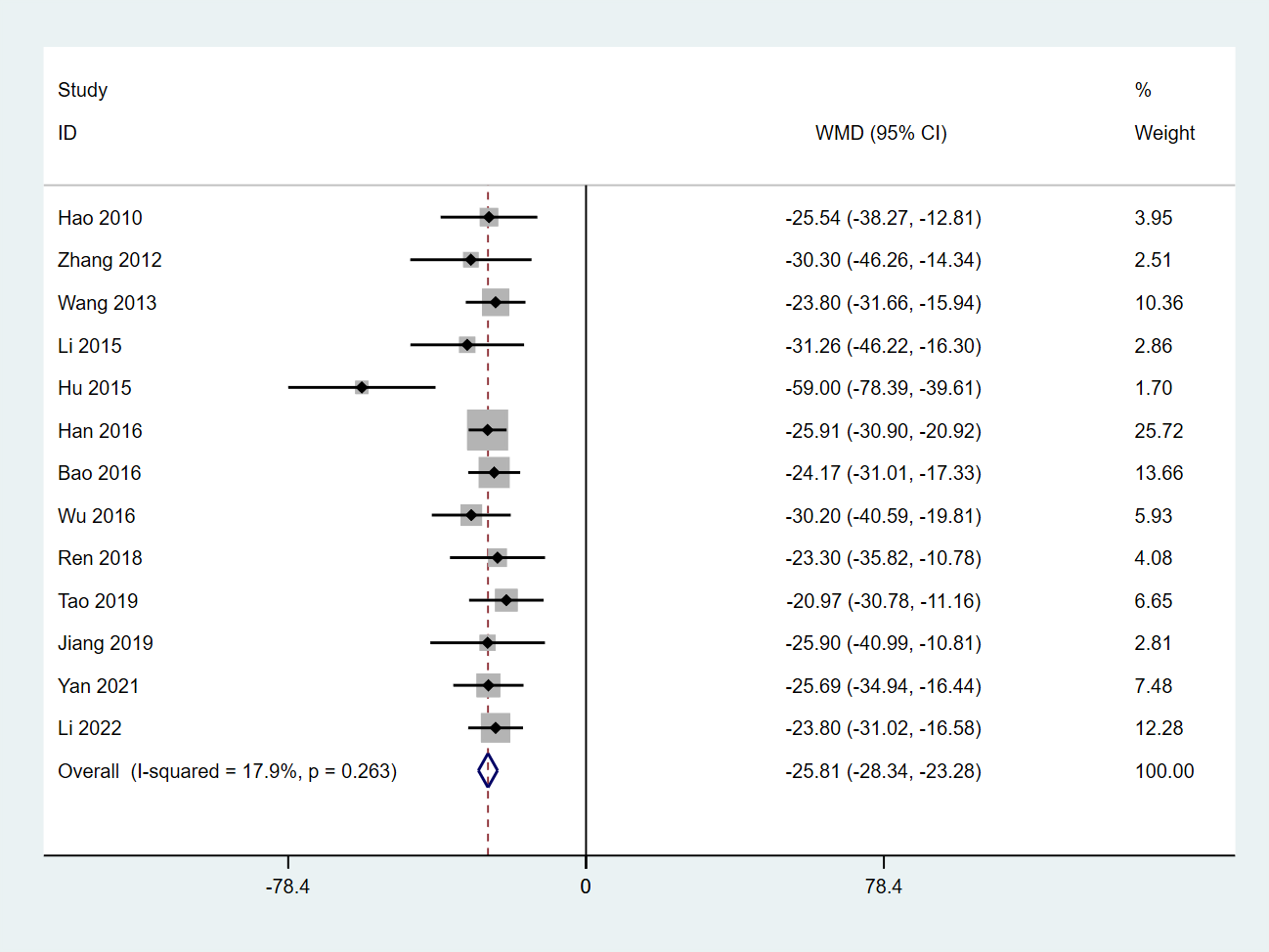

Supplement: Figure documents.zip [file IRNF_A_2499231_SM6438.zip › Figure 4.tif]

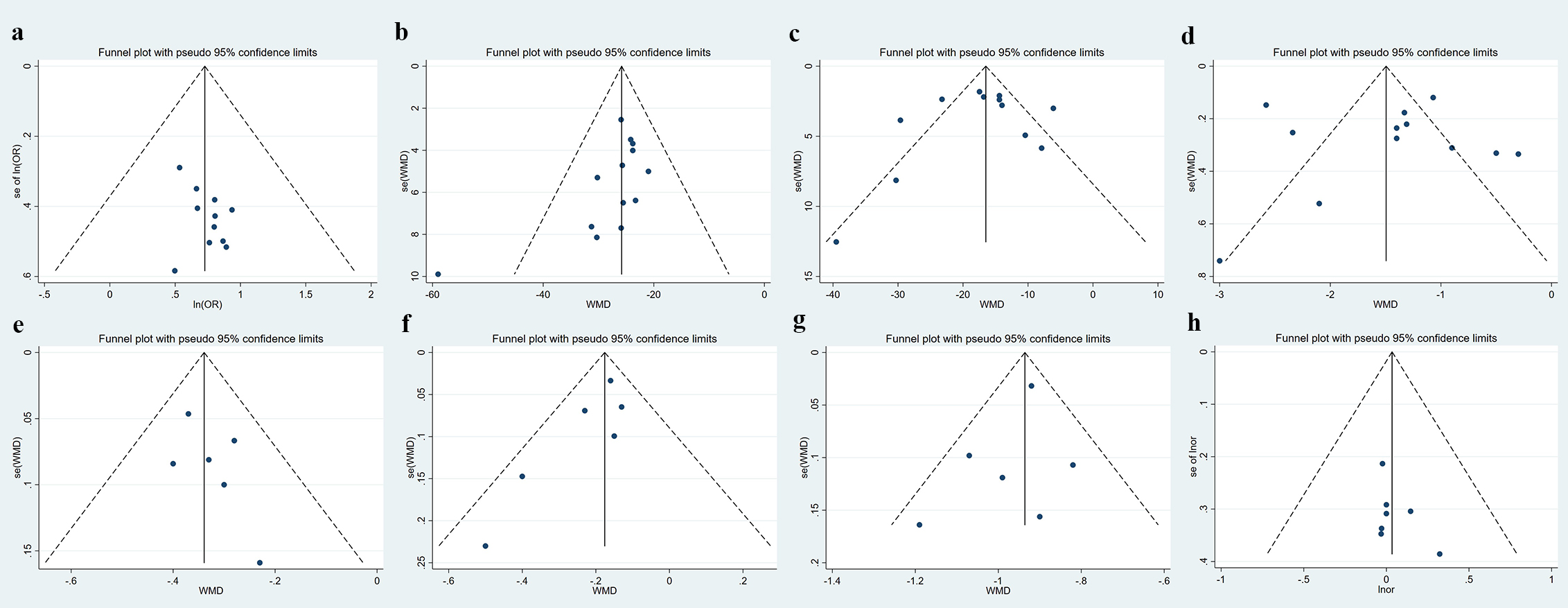

Supplement: Figure documents.zip [file IRNF_A_2499231_SM6438.zip › Figure 5 total.tif]

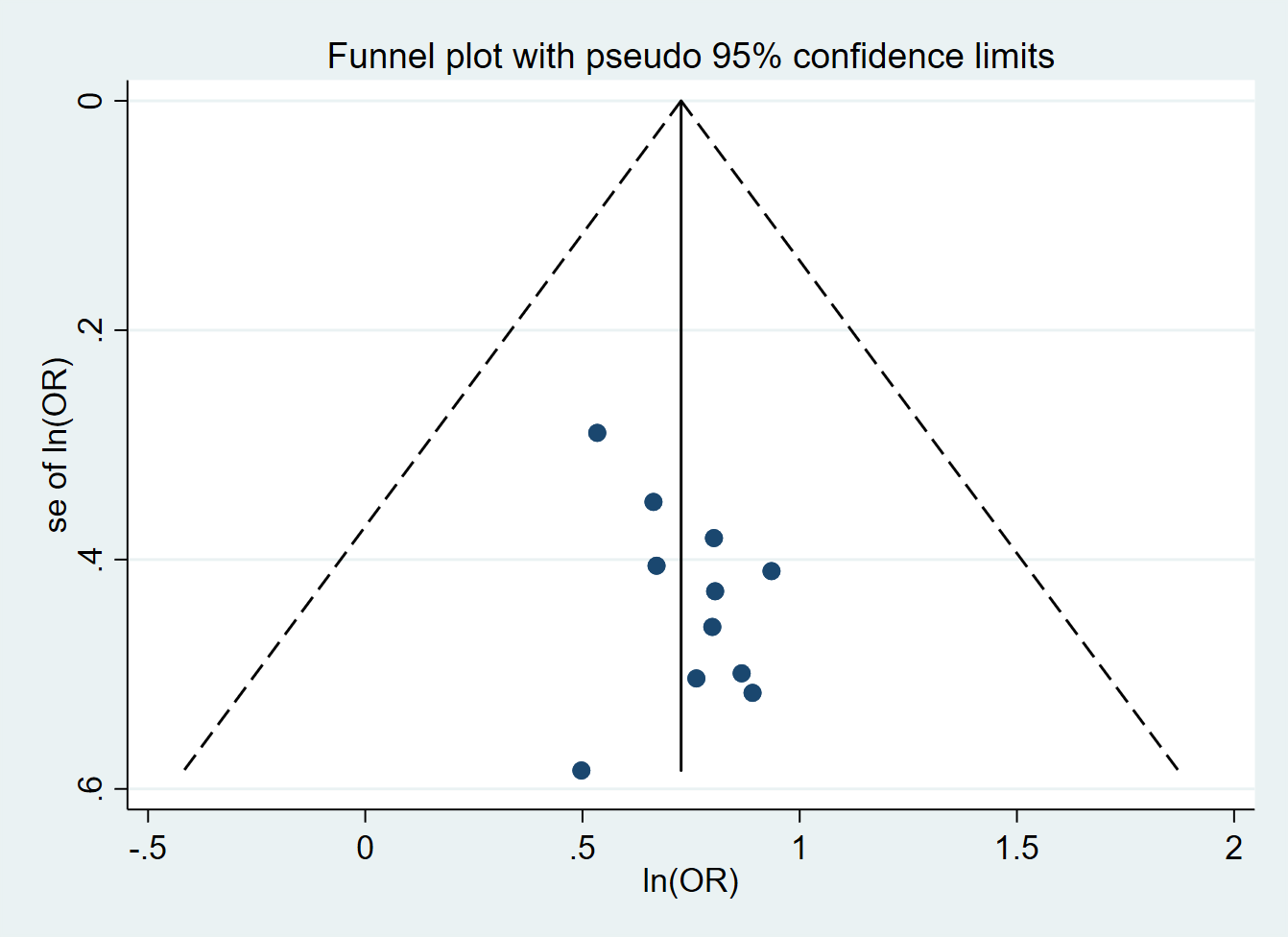

Supplement: Figure documents.zip [file IRNF_A_2499231_SM6438.zip › Figure 5A.tif]

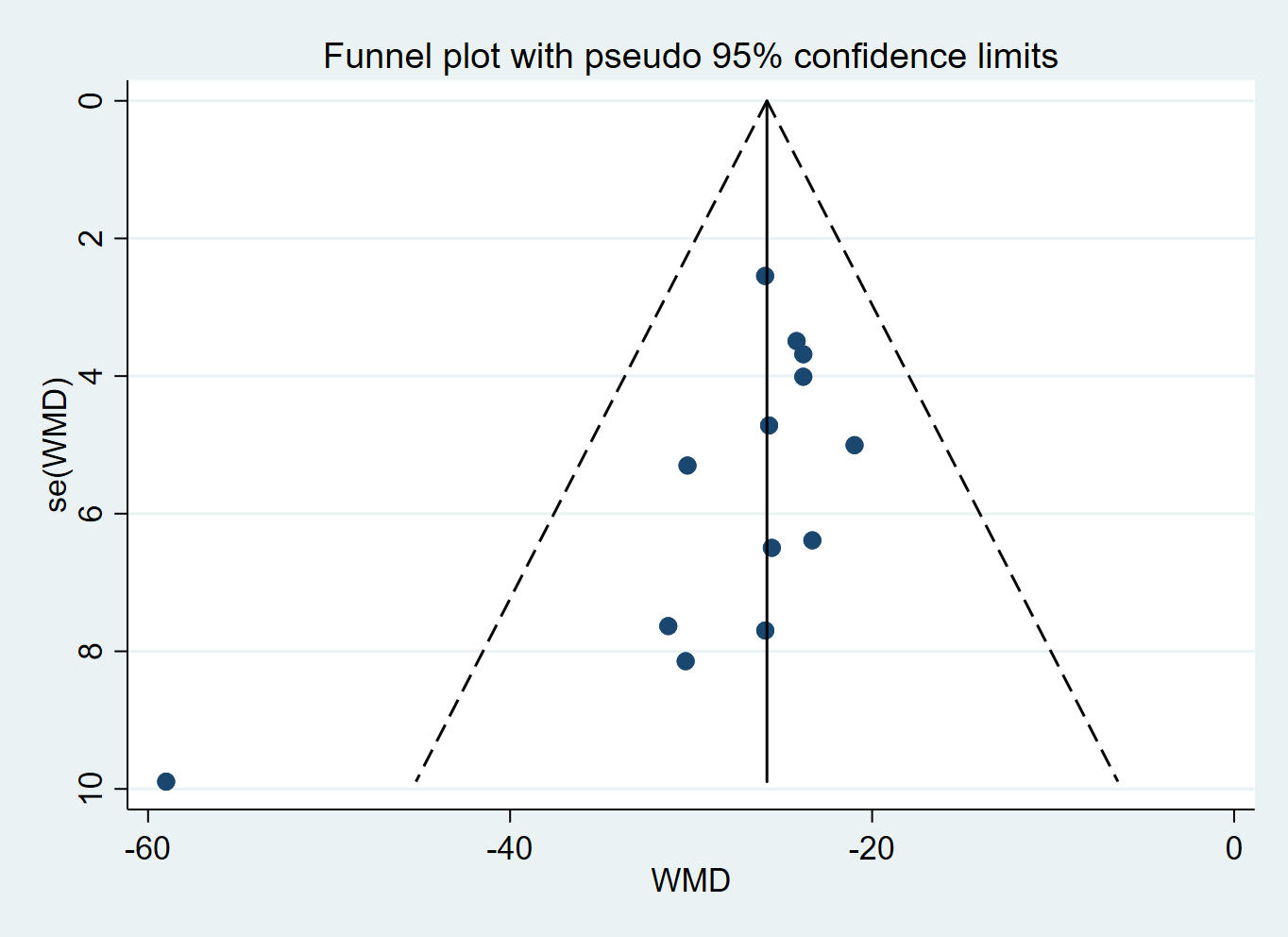

Supplement: Figure documents.zip [file IRNF_A_2499231_SM6438.zip › Figure 5B.tif]

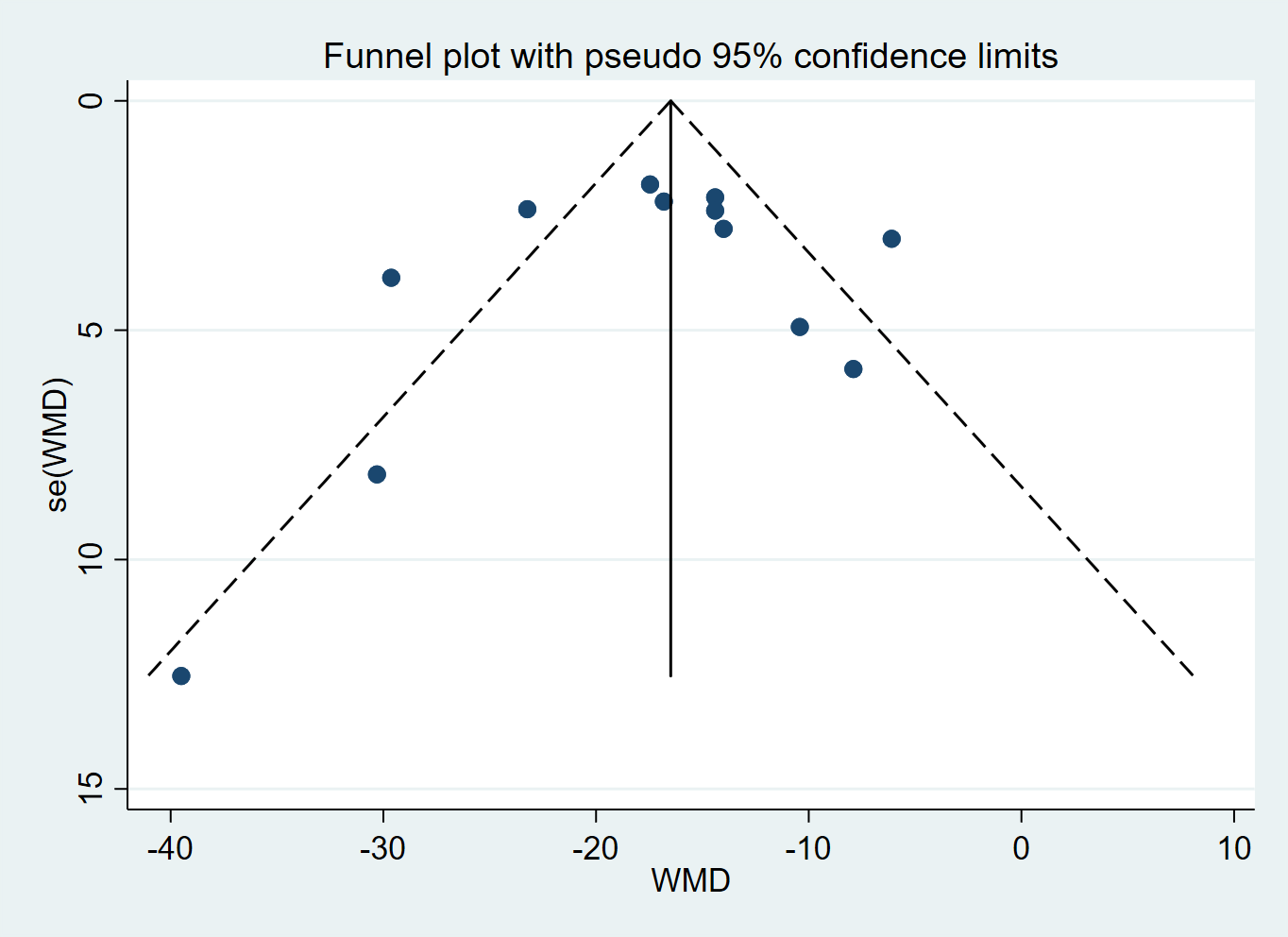

Supplement: Figure documents.zip [file IRNF_A_2499231_SM6438.zip › Figure 5C.tif]

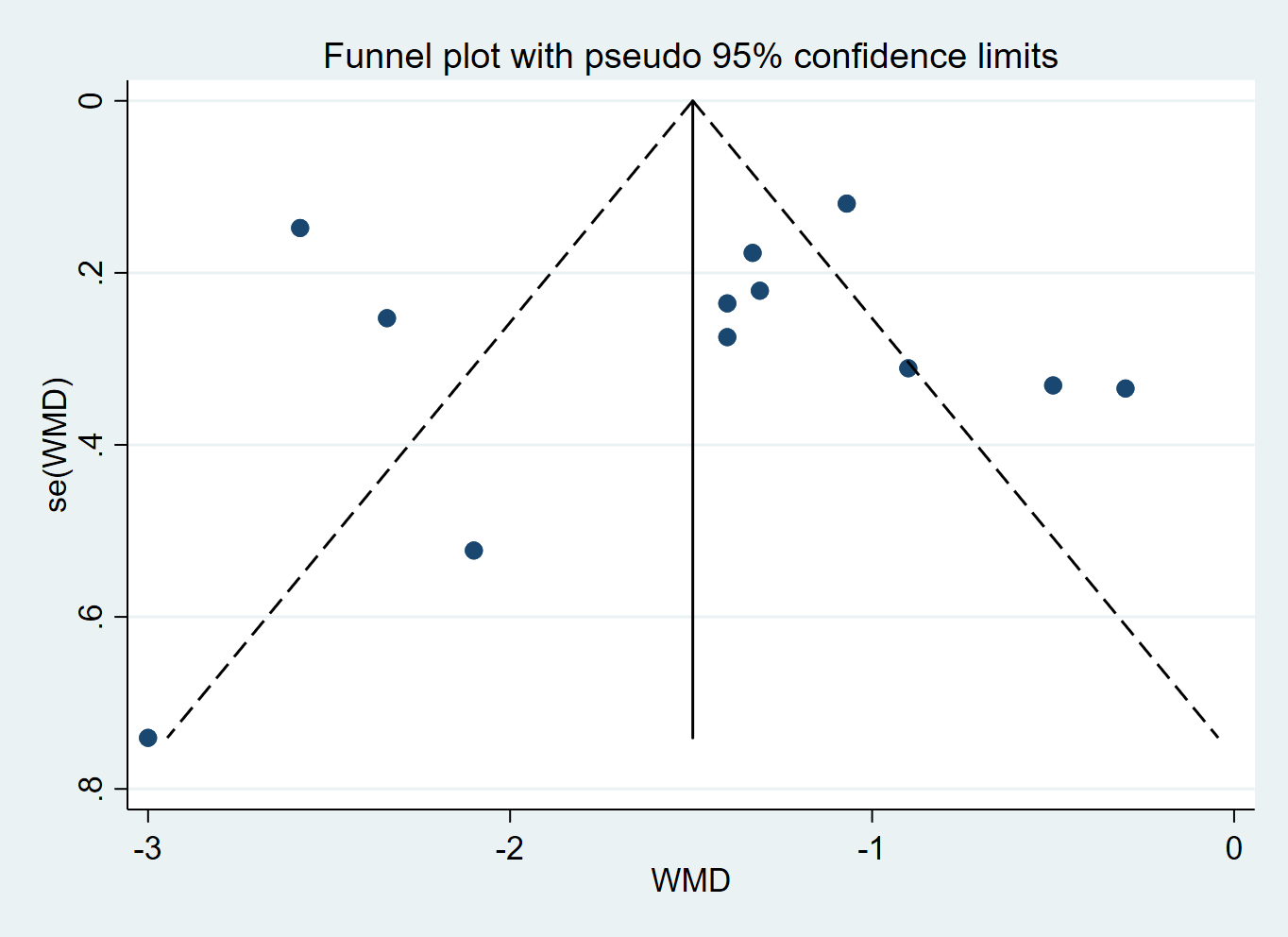

Supplement: Figure documents.zip [file IRNF_A_2499231_SM6438.zip › Figure 5D.tif]

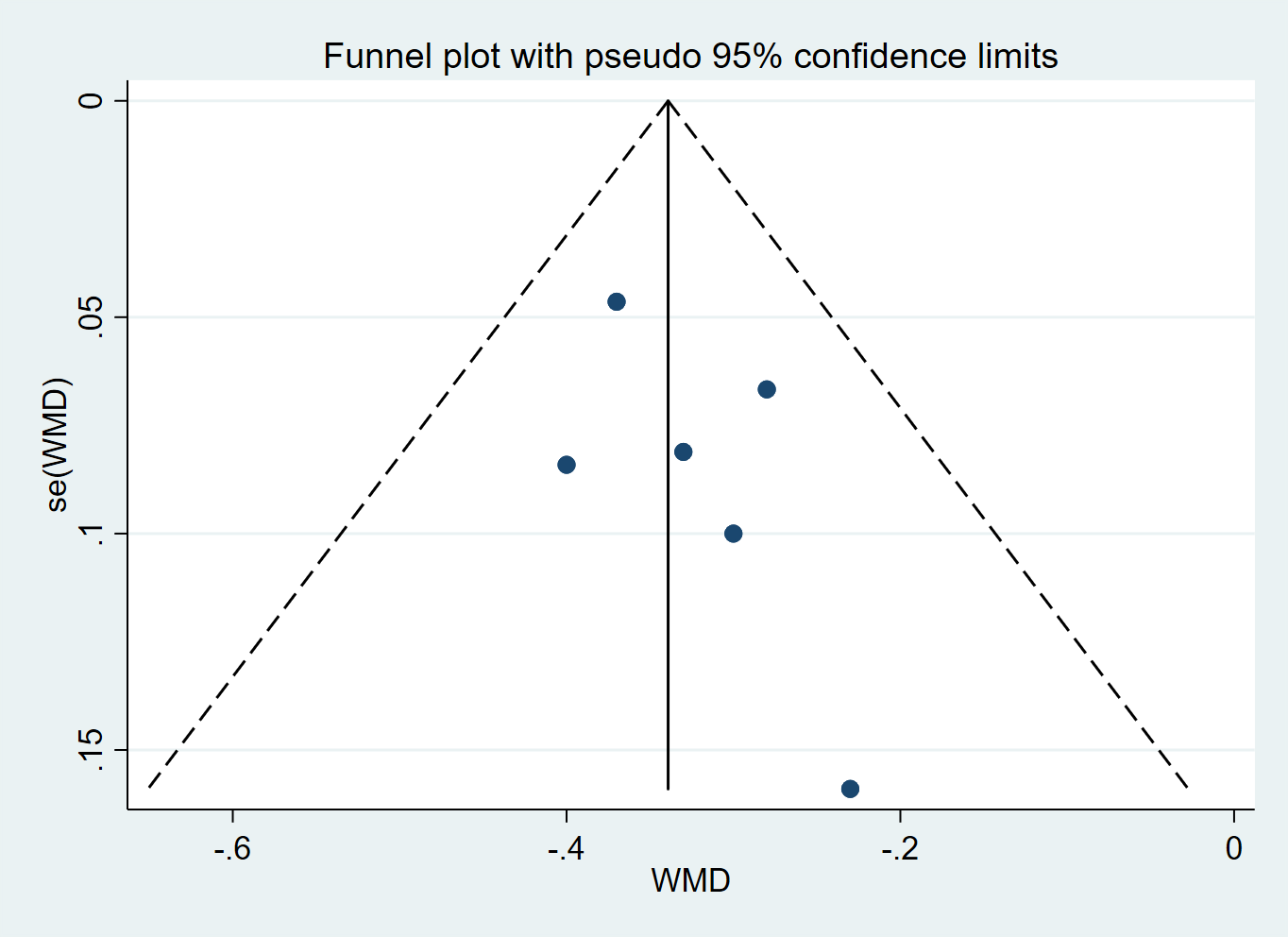

Supplement: Figure documents.zip [file IRNF_A_2499231_SM6438.zip › Figure 5E.tif]

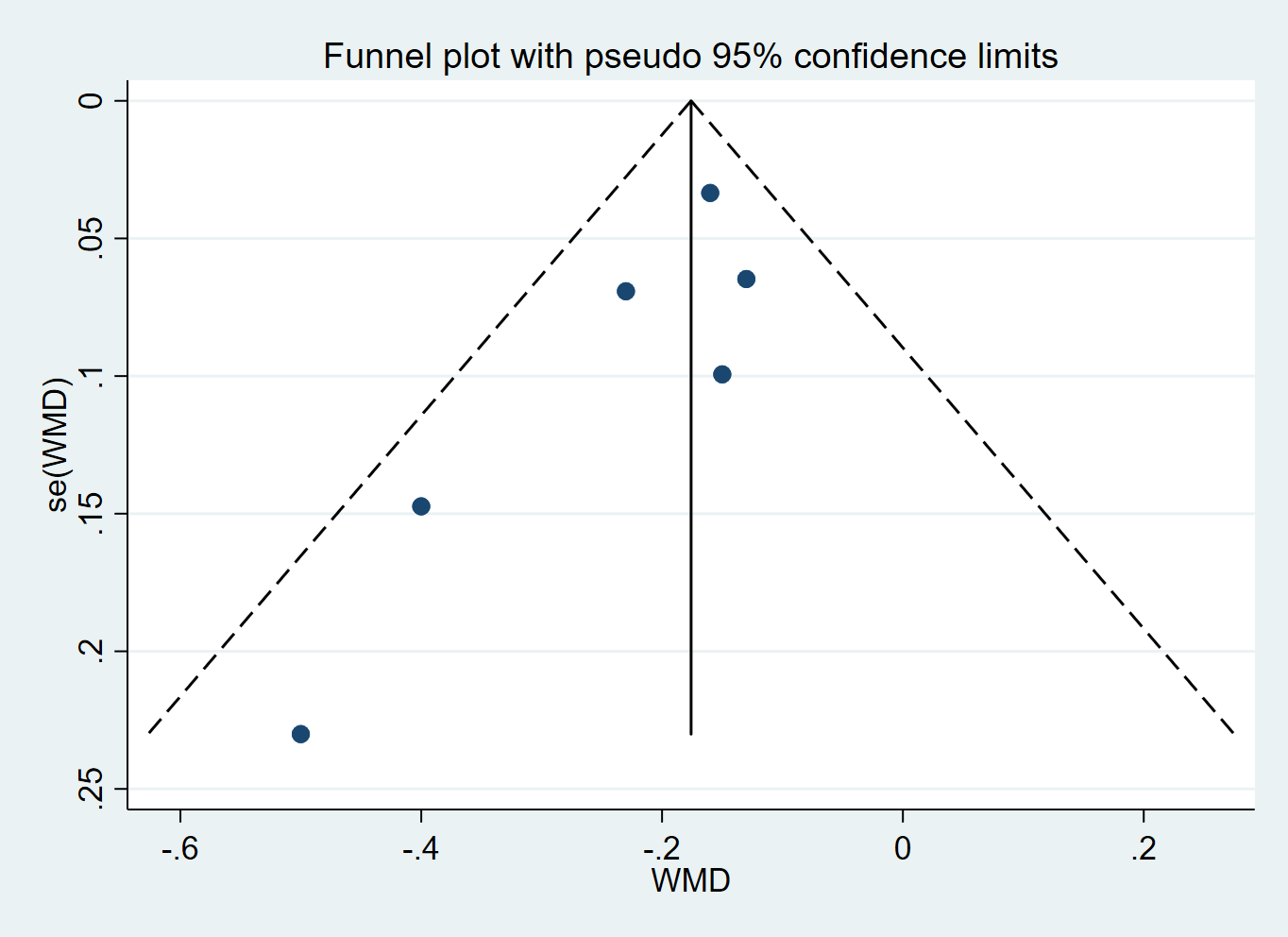

Supplement: Figure documents.zip [file IRNF_A_2499231_SM6438.zip › Figure 5F.tif]

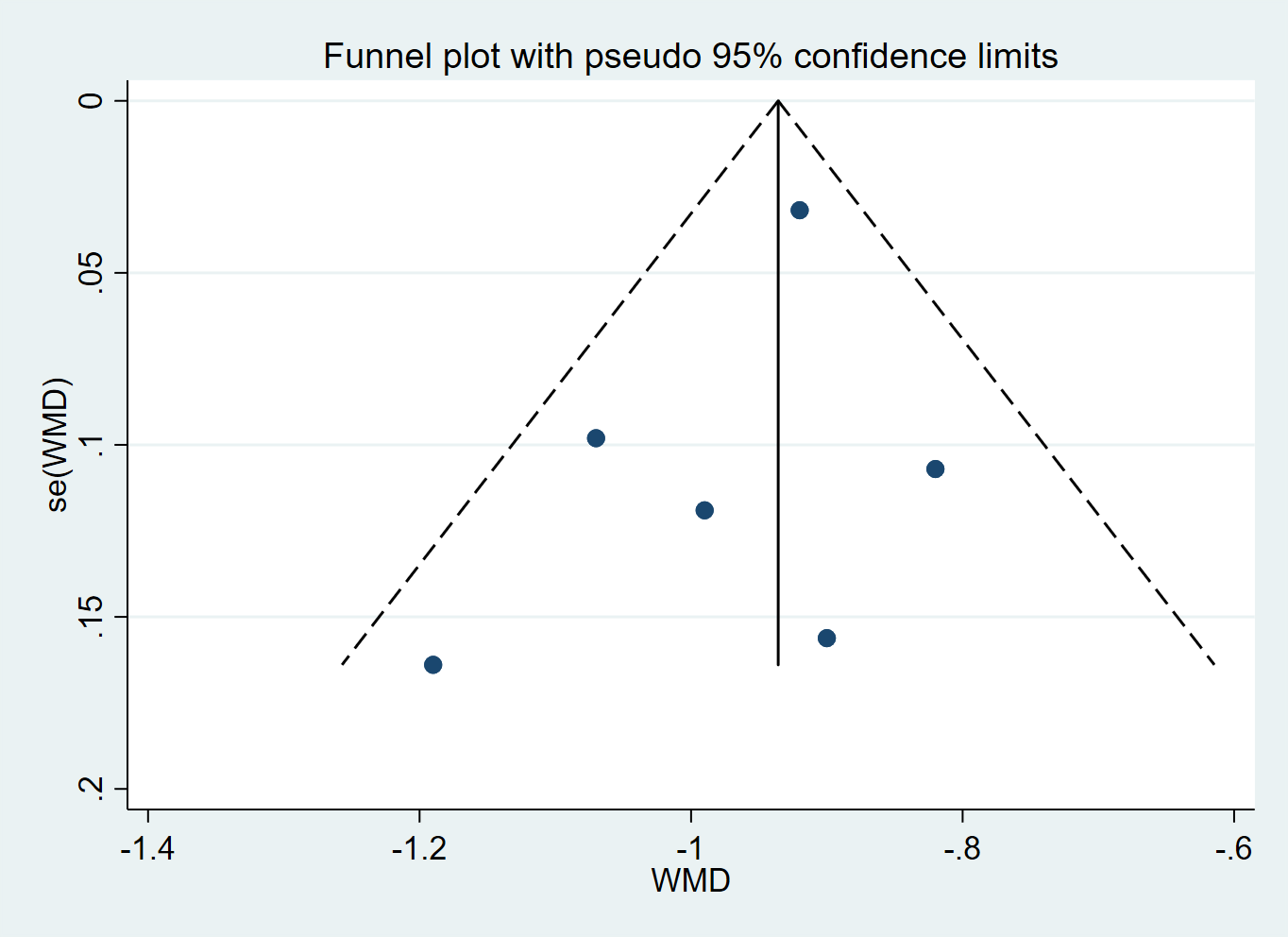

Supplement: Figure documents.zip [file IRNF_A_2499231_SM6438.zip › Figure 5G.tif]

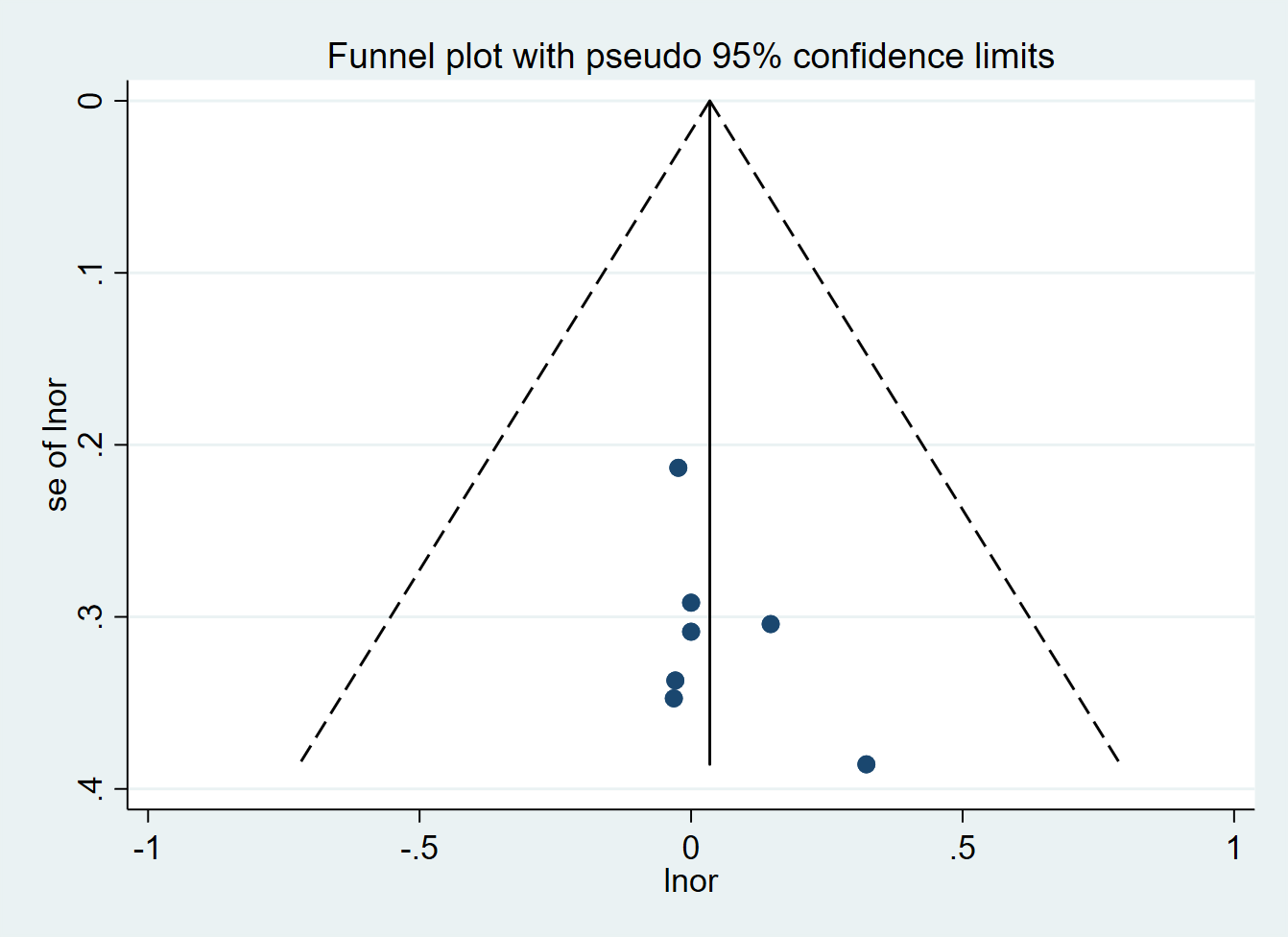

Supplement: Figure documents.zip [file IRNF_A_2499231_SM6438.zip › Figure 5H.tif]

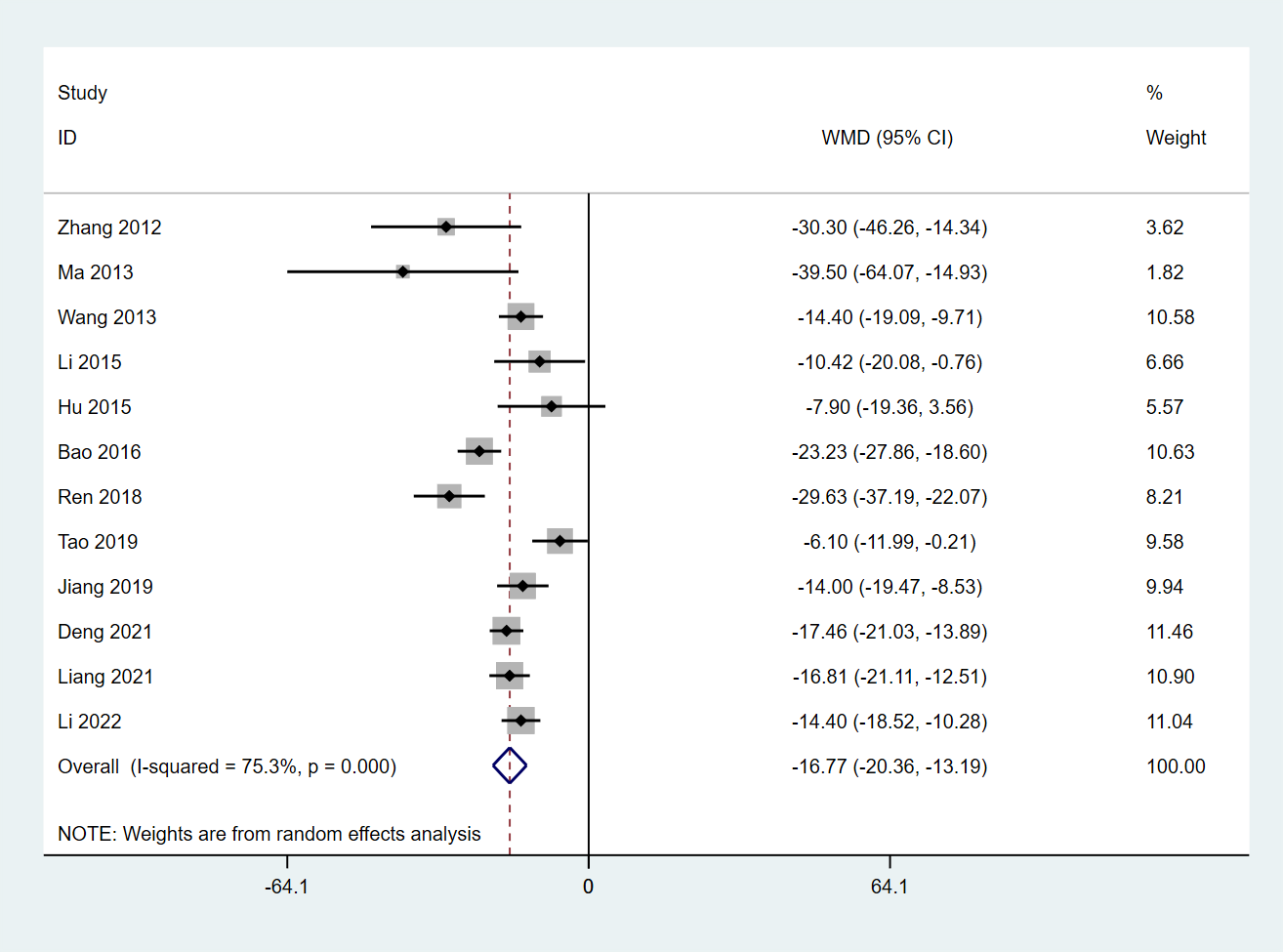

Supplement: Figure Supplementary documents.zip [file IRNF_A_2499231_SM6437.zip › Figure S1.tif]

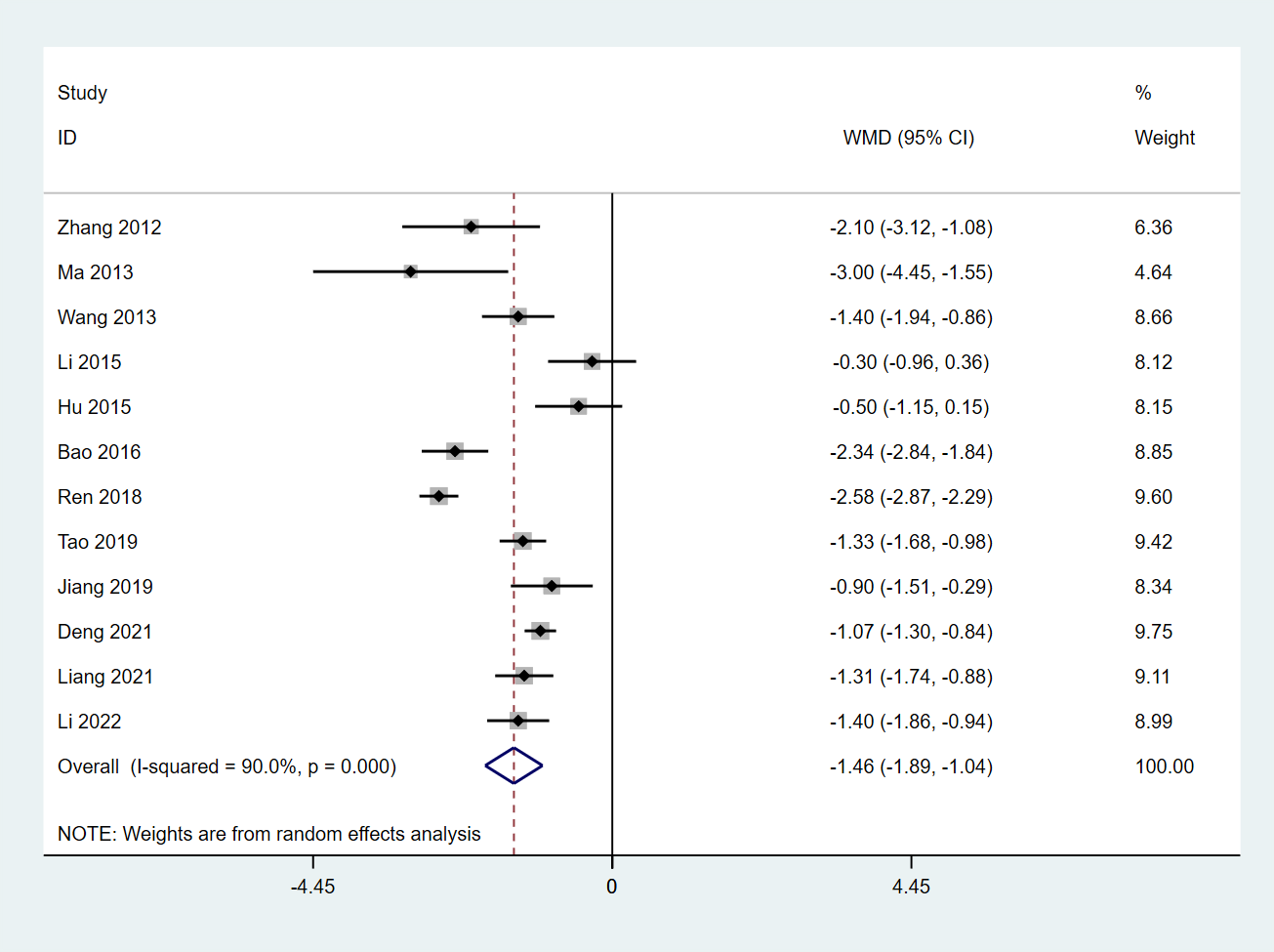

Supplement: Figure Supplementary documents.zip [file IRNF_A_2499231_SM6437.zip › Figure S2.tif]

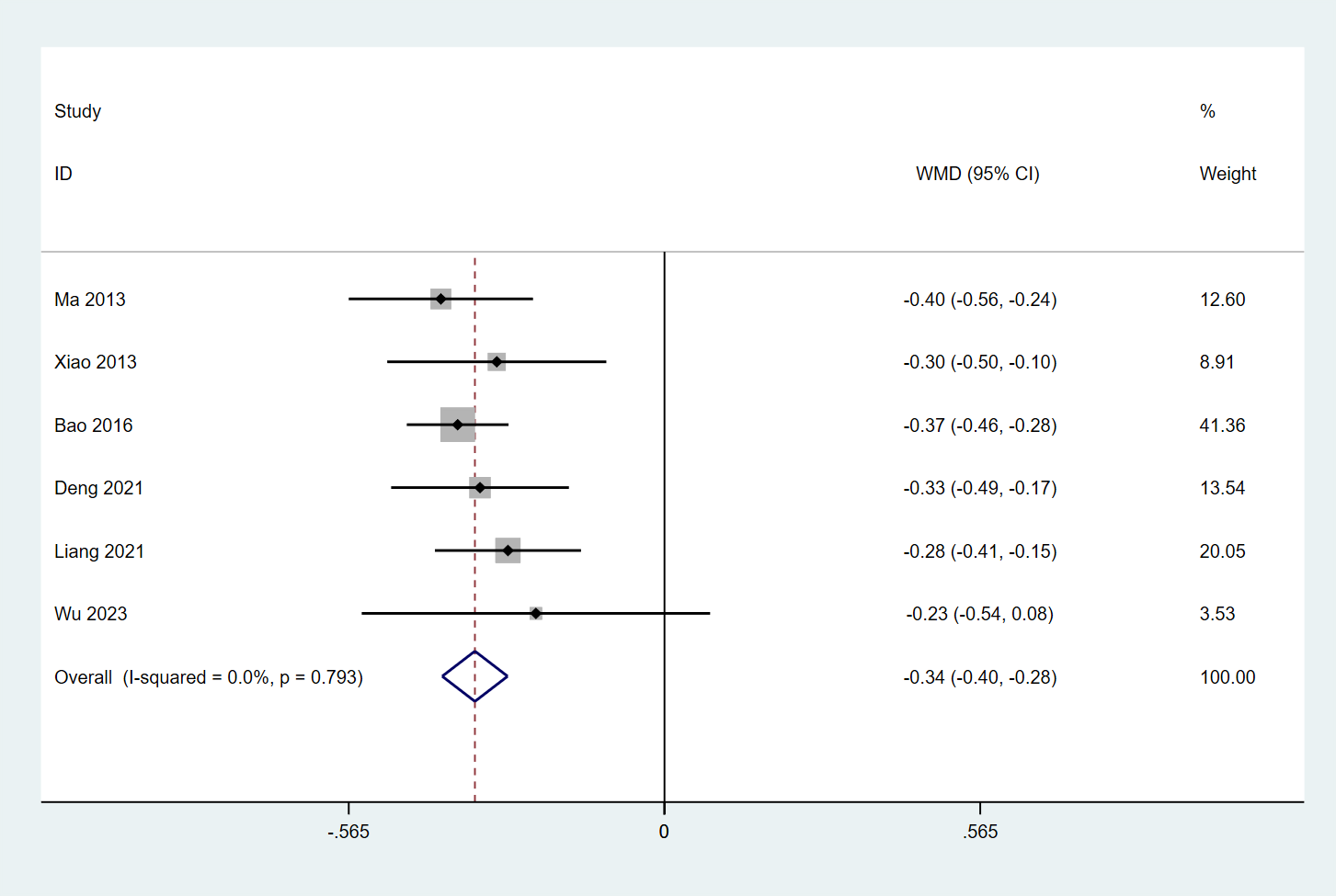

Supplement: Figure Supplementary documents.zip [file IRNF_A_2499231_SM6437.zip › Figure S3.tif]

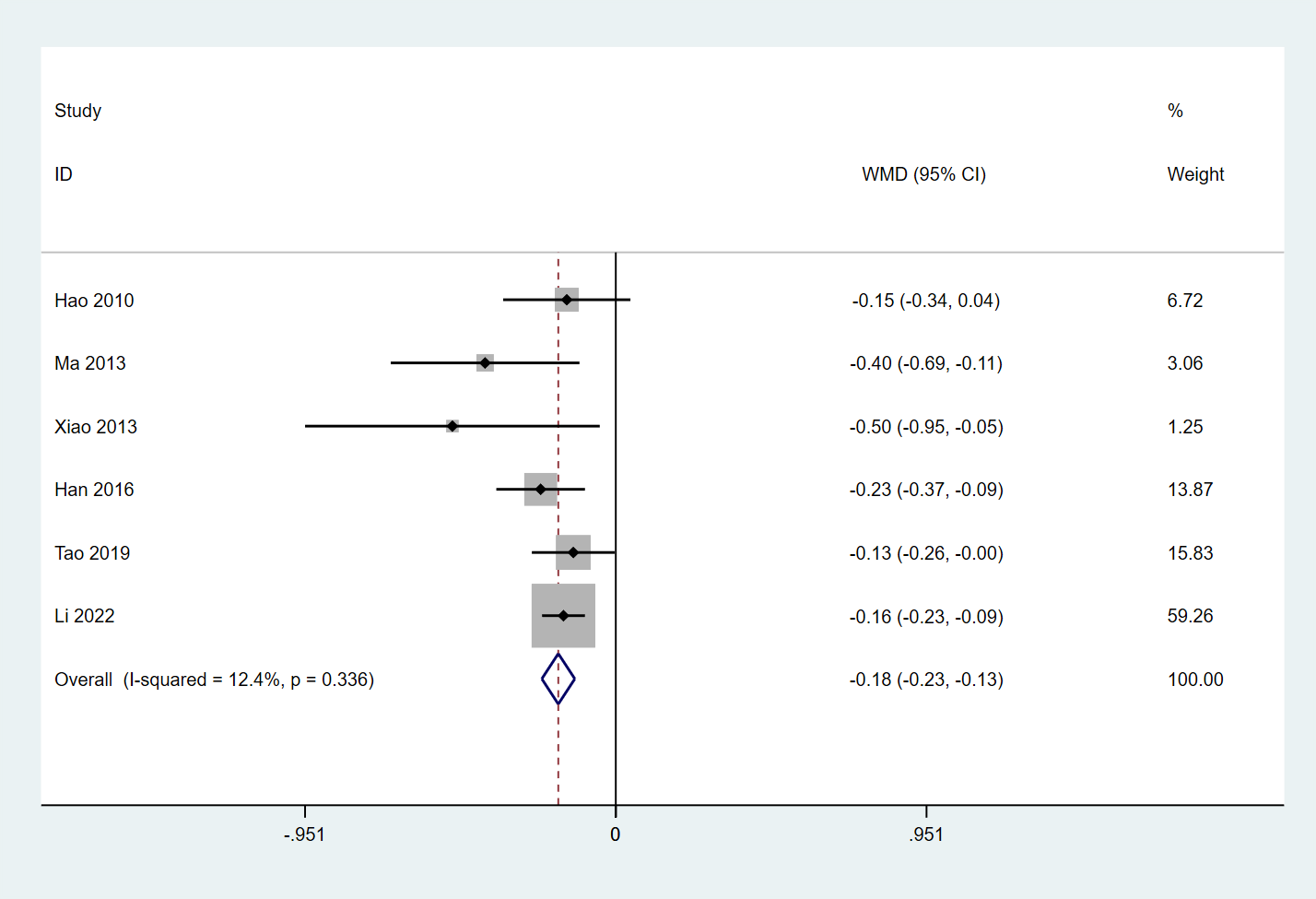

Supplement: Figure Supplementary documents.zip [file IRNF_A_2499231_SM6437.zip › Figure S4.tif]

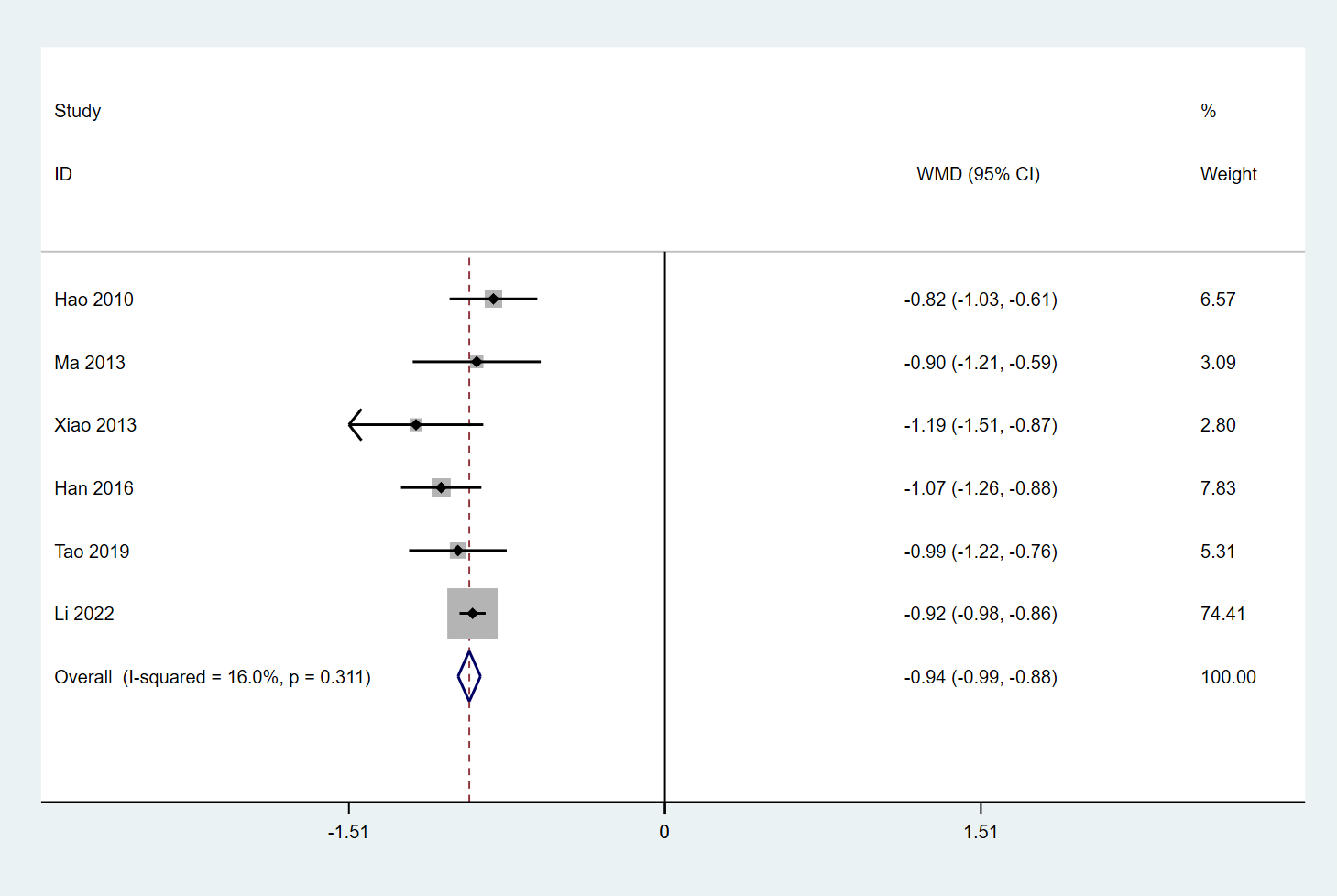

Supplement: Figure Supplementary documents.zip [file IRNF_A_2499231_SM6437.zip › Figure S5.tif]

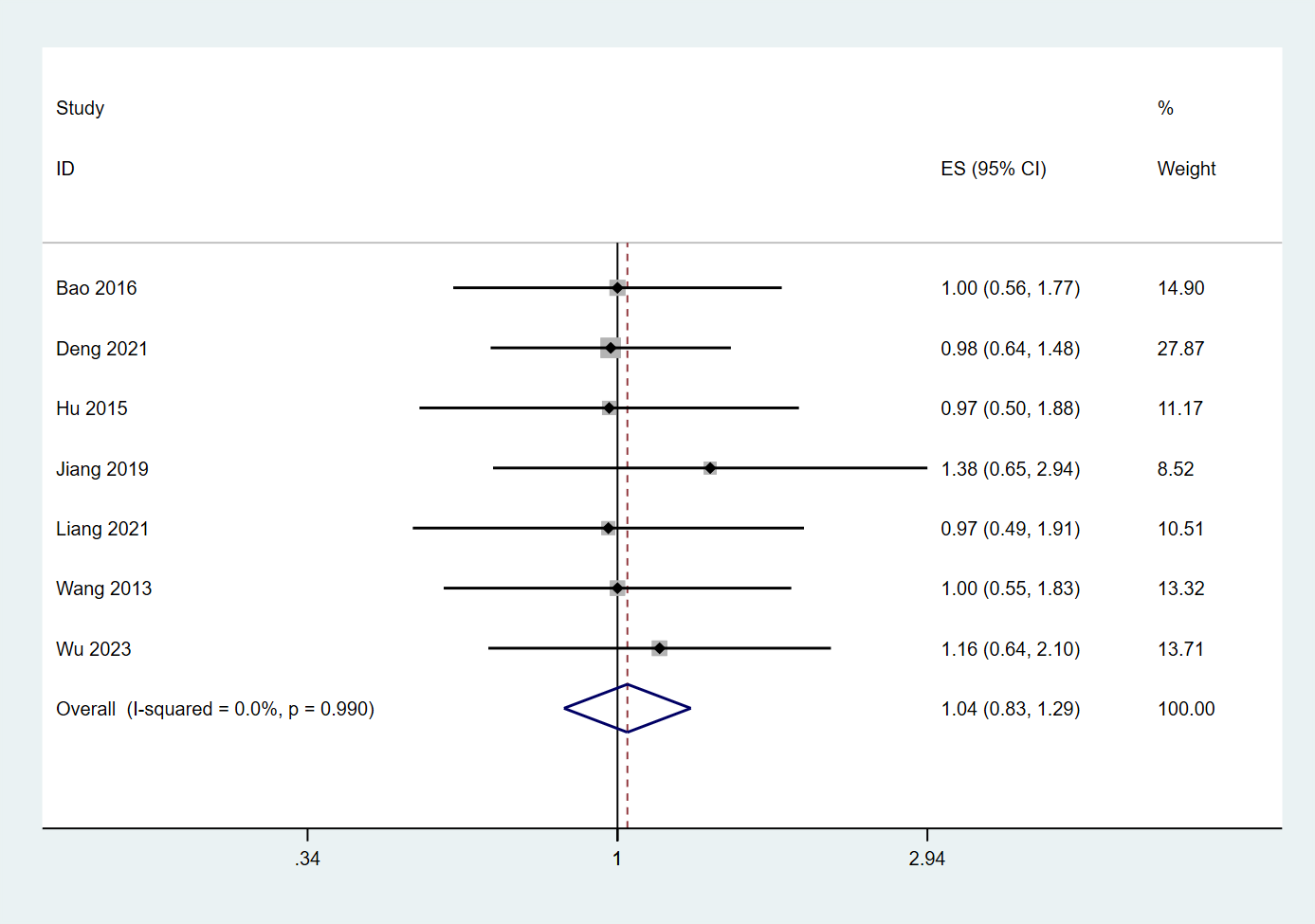

Supplement: Figure Supplementary documents.zip [file IRNF_A_2499231_SM6437.zip › Figure S6.tif]
